# A Three-Ring Circus: Metabolism of the Three Proteogenic Aromatic Amino Acids and Their Role in the Health of Plants and Animals

**DOI:** 10.3389/fmolb.2018.00029

**Published:** 2018-04-06

**Authors:** Anutthaman Parthasarathy, Penelope J. Cross, Renwick C. J. Dobson, Lily E. Adams, Michael A. Savka, André O. Hudson

**Affiliations:** ^1^Thomas H. Gosnell School of Life Sciences, Rochester Institute of Technology, Rochester, NY, United States; ^2^Biomolecular Interaction Centre and School of Biological Sciences, University of Canterbury, Christchurch, New Zealand; ^3^Department of Biochemistry and Molecular Biology, Bio21 Molecular Science and Biotechnology Institute, University of Melbourne, Parkville, VIC, Australia

**Keywords:** tyrosine, phenylalanine, tryptophan, aromatic amino acid biosynthesis, phenylpropanoid metabolism

## Abstract

Tyrosine, phenylalanine and tryptophan are the three aromatic amino acids (AAA) involved in protein synthesis. These amino acids and their metabolism are linked to the synthesis of a variety of secondary metabolites, a subset of which are involved in numerous anabolic pathways responsible for the synthesis of pigment compounds, plant hormones and biological polymers, to name a few. In addition, these metabolites derived from the AAA pathways mediate the transmission of nervous signals, quench reactive oxygen species in the brain, and are involved in the vast palette of animal coloration among others pathways. The AAA and metabolites derived from them also have integral roles in the health of both plants and animals. This review delineates the *de novo* biosynthesis of the AAA by microbes and plants, and the branching out of AAA metabolism into major secondary metabolic pathways in plants such as the phenylpropanoid pathway. Organisms that do not possess the enzymatic machinery for the *de novo* synthesis of AAA must obtain these primary metabolites from their diet. Therefore, the metabolism of AAA by the host animal and the resident microflora are important for the health of all animals. In addition, the AAA metabolite-mediated host-pathogen interactions in general, as well as potential beneficial and harmful AAA-derived compounds produced by gut bacteria are discussed. Apart from the AAA biosynthetic pathways in plants and microbes such as the shikimate pathway and the tryptophan pathway, this review also deals with AAA catabolism in plants, AAA degradation via the monoamine and kynurenine pathways in animals, and AAA catabolism via the 3-aryllactate and kynurenine pathways in animal-associated microbes. Emphasis will be placed on structural and functional aspects of several key AAA-related enzymes, such as shikimate synthase, chorismate mutase, anthranilate synthase, tryptophan synthase, tyrosine aminotransferase, dopachrome tautomerase, radical dehydratase, and type III CoA-transferase. The past development and current potential for interventions including the development of herbicides and antibiotics that target key enzymes in AAA-related pathways, as well as AAA-linked secondary metabolism leading to antimicrobials are also discussed.

## Introduction

**Figure 1:**
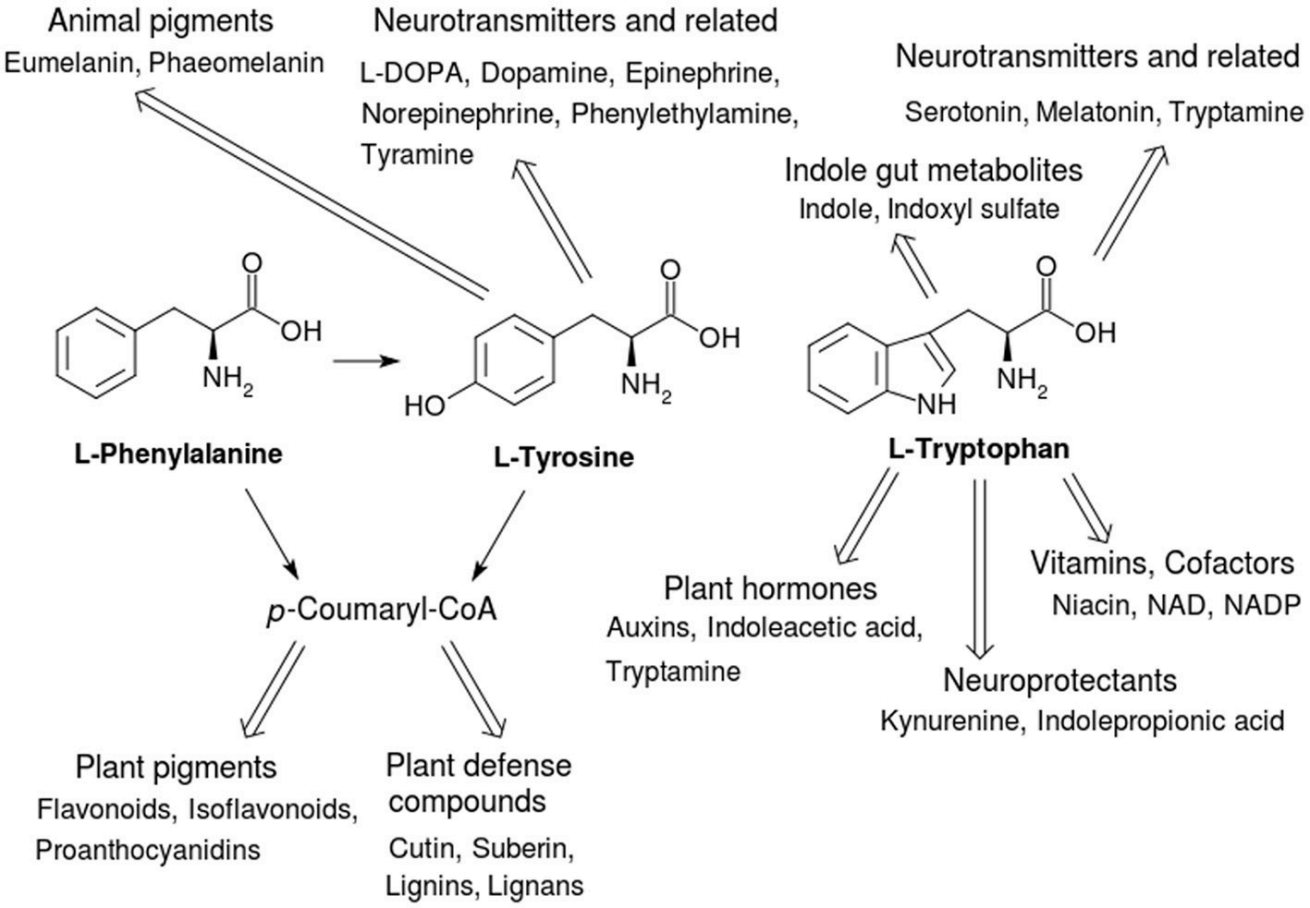
Secondary metabolites derived from the three AAA. The secondary metabolites derived from them classified by their functional roles in plant and animal health. The pathway via *p*-coumaryl-CoA in plants is called the phenylpropanoid pathway and is a major feeder pathway which funnels compounds to fulfill a variety of roles and functions related to health and defense against biotic and abiotic stresses.

The aromatic amino acids (AAA) L-phenylalanine, L-tyrosine and L-tryptophan belong to the family of α-amino acids (AA) ubiquitously involved in the synthesis of proteins. Whereas phenylalanine contains a phenyl group, tyrosine contains a 4-hydroxy phenyl group (making it both an AAA and a hydroxy AA), while tryptophan is aromatic due to its heterocyclic indole ring (Figure 1). The structural and catalytic roles of the AAA in proteins are well known: for example, the AAA aromatic rings stabilize polypeptide structures via π-stacking effects, participate in acid-base catalysis as part of catalytic triads and are involved in charge stabilization and the relay of electrons in the course of electron transfer reactions. The fluorescence changes of tryptophan have been widely used as an indicator of changes in enzyme structure and function. Tyrosine is a radical initiator/storage moiety in enzyme catalysis and has additional regulatory roles since its hydroxyl group can be phosphorylated. Tyrosine can in addition act as a nucleophile in some enzymatic reactions, for example the trans-sialidase reaction in *Trypanosoma cruzi*, whereby the enzyme catalyzes the transfer of a sialic acid group via a covalent aryl glycoside intermediate (Watts et al., 2003). Due to its hydroxy group, tyrosine is able to coordinate metals such as iron for example in the iron-storage protein ferritin, where it may have an additional redox role in the di-iron center (Ebrahimi et al., 2013). The cross-linking of proteins via tyrosyl groups can also occur through the hydroxyl functional group; for example in the cuticle of *Caenorhabditis elegans*, collagen and other structural proteins are linked by di- and tri-tyrosyl bridges (Edens et al., 2001).

Not only are the AAA important for these functions in proteins, but AAA biosynthesis and degradation also act as starting points for a large variety of secondary metabolites that have integral and key roles in plants and animals (Figure 1). Here we examine some anabolic and catabolic aspects of AAA metabolism in the context of plant and animal health, with an emphasis on enzyme function and structure. Table 1 shows a list of important AAA-linked enzymes discussed in this article.

**Table 1:** List of major enzymes discussed in this article, their biological roles and properties; entries with unknown structures mentioned in bold.

**Enzyme name**	**Section name**	**Occurrence**	**Biological role**	**Properties**
Anthranilate synthase (AS)	Anthranilate synthase (AS)	Microbes including pathogens, plants	Tryptophan biosynthesis	Two subunits AS-I and AS-II; composition not universal, nucleophilic substitution by ammonia and amidotransferase activity
Tryptophan synthase	Indole glycerol phosphate synthase (IGP synthase)	Microbes including pathogens, plants	Tryptophan biosynthesis	α_2_β_2_ tetramer. α subunit generates indole from IGP by retro-aldol reaction. Indole chaneled into the second active site, present in the B or β subunit. second active site involves a PLP Schiff base mechanism
Tyrosine aminotransferase (TAT)	Phenylalanine and tyrosine biosynthesis	Microbes	Phenylalanine and tyrosine biosynthesis	PLP-containing, homodimeric with small and large domains in each monomer
	Tyrosine aminotransferase in plants	Plants	Links phenylalanine and tyrosine to secondary metabolism	PLP-containing **Structure of plant enzyme unsolved**
	AAA catabolism in animals	Animals	Tyrosine degradation	PLP-containing
Dopachrome converting enzymes	Dopachrome converting enzymes – DCT and DCDT			
(1) DCT		Mammals	Melanin biosynthesis	Binuclear zinc center
(2) DCDT		Insects, other invertebrates	Melanin biosynthesis	**Structure unknown**
Phenylalanine hydroxylase (PheOH)	AAA hydroxylases (AAAH)	Animals	Synthesis of tyrosine and catcholamines	Pterin dependent, catalytic iron, substrate allostery regulates
Tryptophan hydroxylase (TyrOH)	AAA hydroxylases (AAAH)	Animals	Synthesis of catecholamines	Pterin dependent, catalytic iron, catecholamine binding regulates
Aromatic amino acid decarboxylase (AADC)	AAA decarboxylase (AADC) and other enzymes	Animals	Synthesis of neurotransmitters	PLP-dependent decarboxylase
		Plants	Synthesis of defense compounds	PLP-dependent decarboxylase
3-Aryllactate dehydratase	Radical dehydratases and the 3-aryllactate pathway	Anaerobic gut bacteria	AAA fermentation in mammalian gut	Radical iron-sulfur enzyme forming complex with activator enzyme and Type III CoA-transferase
TypeIII CoA-transferase	Type III CoA-transferase	Anaerobic gut bacteria	AAA fermentation in mammalian gut	Ternary complex mechanism without covalent enzyme-CoA intermediate

## The biosynthesis of AAA

The shikimate pathway represents the common seven step biosynthetic route to all three AAA. From chorismate onwards, AAA biosynthesis diverges, with distinct pathways for each of the AAA and further variations between plants and microorganisms.

### The shikimate pathway

The shikimate pathway is present in bacteria, fungi, plants and algae, as well as some parasitic protozoans. This pathway does not occur in animals and, therefore, animals must obtain the AAA as essential nutrients from their diet. The 3-carbon sugar phosphoenol pyruvate (PEP) and the 4-carbon sugar erythrose-4-phosphate (E4P) are the initial precursors which are condensed by (3-deoxy-*D*-arabinoheptulosonate 7-phosphate (DAHP) synthase (DAHPS) (EC 2.5.1.54) with the hydrolysis of phosphate into the 7-carbon compound DAHP (Entus et al., 2002). In plants, DAHPS is Mn^2+^- and thioredoxin-dependent and thus links the flow of carbon into this pathway with the flow of electrons from photosystem I (Entus et al., 2002). This step constitutes a key regulatory step in the pathway, with many, but not all, DAHPS enzymes known to have a variety of regulatory domains that allow feedback inhibition by tyrosine, phenylalanine or tryptophan or by combinations of multiple AAAs (Cross et al., 2011, 2013; Blackmore et al., 2013). In the second step, the 7-carbon product synthesized in the first step is oxidized by 3-dehydroquinate synthase (DHQS) (EC 4.2.3.4) with the help of NAD^+^, whereby the elimination of the phosphate leads to a cyclic product. This product (3-dehydroquinate) undergoes the loss of a water molecule, leading to 3-dehydroshikimate. The next step is the NADPH-dependent reduction of 3-dehydroshikimate into shikimate. These two steps are catalyzed by the bi-functional enzyme 3-dehydroquinate dehydratase/shikimate 5-dehydrogenase (DHQ/SDH) (EC 4.2.1.10 and EC 1.1.1.25). Shikimate is then phosphorylated at the 3-position by shikimate kinase (SK) (EC 2.7.1.71) using one ATP per shikimate molecule. The coupling of shikimate to phosphoenol pyruvate to form 5-enolpyruvylshikimate-3-phosphate (EPSP) is catalyzed by EPSP synthase (EPSPS) (EC 2.5.1.19). Finally, the transformation of EPSP to chorismate involves the elimination of phosphate and ring oxidation by chorismate synthase to yield chorismate.

### Phenylalanine and tyrosine biosynthesis

**Figure 2:**
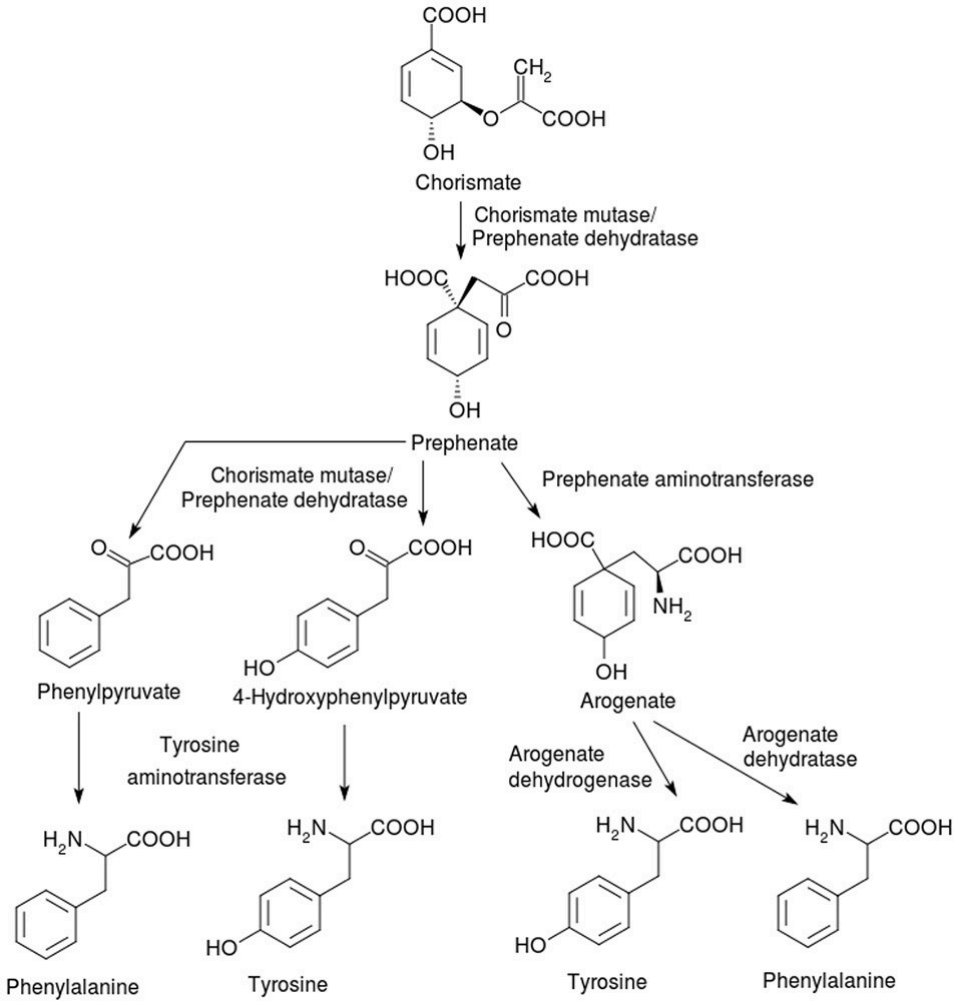
Synthesis of the phenylalanine and tyrosine from the intermediate chorismate. Animals do not have complete pathways for the three AAA, which are anabolized *de novo* only by bacteria, other microorganisms and plants. The starting point for all three AAA is the shikimate pathway (not shown) which generates the common intermediate chorismate. The pathways on the left involving the bifunctional enzyme chorismate mutase/prephenate dehydratase (PheA or TyrA) and tyrosine aminotransferase (TyrB) are found in bacteria, while the ones on the right (via arogenate) occur in plants that use the enzymes prephenate aminotransferase, arogenate dehydrogenase and arogenate dehydratase.

The bacterial synthesis of phenylalanine and tyrosine, starting from chorismate, is shown in Figure 2. Chorismate mutase, also known as hydroxyphenylpyruvate synthase or chorismate pyruvatemutase, is the isomerase enzyme involved and catalyzes the committed step of phenylalanine and tyrosine, namely the formation of prephenate. In the phenylalanine anabolic pathway, the bifunctional enzyme chorismate mutase (EC 5.4.99.5)/prephenate dehydratase (EC 4.2.1.51), which is usually encoded by the *pheA* gene, rearranges chorismate to prephenate and converts the latter into phenylpyruvate. In tyrosine biosynthesis, the bifunctional enzyme chorismate mutase/prephenate dehydrogenase (EC 5.4.99.5) encoded by the *tyr*A gene, transforms chorismate into 4-hydroxyphenylpyruvate, rather than phenylpyruvate.

Both phenylalanine and tyrosine biosynthesis involve the tyrosine aminotransferase (TAT) (EC 2.6.1.5), which is dependent on pyridoxal-5-phosphate (PLP) and is encoded by the *tyrB* gene (Prabhu and Hudson, 2010), as the final enzyme, whereby glutamate acts as the amino group donor. Although the Integrated Microbial Genomes (IMG) database lists aromatic aminotransferases as necessary genes for AAA biosynthesis, genes contributing to the synthesis of other amino acids may perform aminotransferase functions in phenylalanine and tyrosine biosynthesis in many bacteria (Pittard and Yang, 2008). The involvement of other aminotransferases, such as the branched chain aminotransferase IlvE (EC 2.6.1.42) and the aspartate aminotransferase AspC (EC 2.6.1.1) in tyrosine and phenylalanine anabolism is known owing to their promiscuous substrate specificity, which allows an overlap with TyrB activity (Mavrides and Orr, 1975; Gelfand and Steinberg, 1977; Whitaker et al., 1982). Thus, multiple aminotransferases with overlapping substrate seems to be a bacterial strategy evolved to enable nutritional flexibility under various growth conditions. In fact, directed evolution of aspartate aminotransferase to TAT in bacteria could be accomplished without the loss of the original aspartate aminotransferase function (Rothman and Kirsch, 2003), suggesting that enzymes performing both functions likely arose from the same ancestor. Also, in some cases genes annotated as aromatic aminotransferases in genome databases have been found to encode enzymes with other functions. For example, the putative aromatic aminotransferase CgAro8p in the fungus *Candida glabrata* was shown to participate in histidine degradation (Brunke et al., 2014).

The phenylalanine and tyrosine biosynthetic pathway in plants is distinct from that in bacteria and fungi, but also proceeds via chorismate (Bender, 2012). The major difference is that the plant pathway involves an aminotransferase reaction at the penultimate, rather than the ultimate step. Prephenate is generated from chorismate by chorismate mutase (EC 5.4.99.5). The next step is a glutamate-dependent aminotransferase reaction catalyzed by prephenate aminotransferase (EC 2.6.1.79), generating arogenate from prephenate. Arogenate is a branching point; arogenate oxidation and decarboxylation by the oxidoreductase enzyme arogenate dehydrogenase (EC 1.3.1.43) leads to the formation of tyrosine; arogenate is converted by the lyase enzyme arogenate dehydratase (EC 4.2.1.91) (Buchanan et al., 2000) into phenylalanine. The site of the biosynthesis of all the AAA in plants is within the plastid (Bickel et al., 1978). While chorismate mutases have been reported from the cytosol in several plant species (d'Amato et al., 1984; Benesova and Bode, 1992; Eberhard et al., 1996), the presence of subsequent enzymes in the pathway is under debate. Recent work has demonstrated the localization of the key enzymes arogenate dehydrogenase (tyrosine biosynthesis) and arogenate dehydratase (phenylalanine biosynthesis) to the plastids in Arabidopsis (Rippert et al., 2009).

#### Tyrosine aminotransferase in plants

Tyrosine aminotransferase (TAT) (EC 2.6.1.5) is an essential enzyme in the biosynthesis of tyrosine via the bacterial pathway as discussed in section Phenylalanine and tyrosine biosynthesis. The genome of the model plant *Arabidopsis thaliana* contains 44 putative aminotransferase or aminotransferase-like genes, out of which seven are annotated as TAT. Since the plant pathway via arogenate fulfills the biosynthetic needs for generating phenylalanine and tyrosine, the TAT candidates could be expected to play other roles. Of the seven potential Arabidopsis genes encoding for potential TAT enzymes, At5g36160 was deemed “putative” in public databases. The corresponding enzyme was identified and characterized as a TAT which could use both tyrosine and phenylalanine as substrates (Prabhu and Hudson, 2010). Interestingly, although the amino acid sequence of this enzyme bears only 7.6% identity to the *E. coli* TAT ortholog, the At5g36160 gene was able to functionally complement an *E. coli* TAT mutant auxotrophic for both tyrosine and phenylalanine. The aminotransferase encoded by this gene was predicted to be localized in the cytoplasm by the use of sub-cellular localization prediction algorithms (Prabhu and Hudson, 2010). Even though TAT activity has been demonstrated for certain orthologs, it is not clear what the role/s of the enzymes are regarding AAA metabolism especially since aminotransferases are known to be promiscuous. AAA biosynthesis in plants occurs in the plastid, suggesting that the cytoplasmic aminotransferase encoded by the locus tag At5g36160 is not involved in anabolism of tyrosine and phenylalanine, but rather in the catabolism of tyrosine and phenylalanine in order to shuttle metabolites involved in anabolism through the phenylpropanoid pathways.

TAT enzymes have also been implicated in diverting phenylalanine and tyrosine for other secondary metabolite pathways, such as the production of antioxidants that scavenge free radicals and protect plants from various stresses. Examples include tocopherols (Lopukhina et al., 2001; Höllander-Czytko et al., 2005) and rosmarinic acid (De-Eknamkul and Ellis, 1987; Xiao et al., 2009). In *Papaver somniferum*, TAT was suggested to be involved in the synthesis of benzylisoquinoline, which is itself rerouted to produce many alkaloids such as papaverine, codeine, morphine and apomorphine (Lee and Facchini, 2011). *Pseudomonas syringae* is a plant pathogen which produces the phytotoxin coronatine (Gnanamanickam et al., 1982). It was demonstrated that the TAT isoform annotated by the locus tag At4g23600 from *A. thaliana* was regulated by coronatine (Lopukhina et al., 2001).

**Figure 3:**
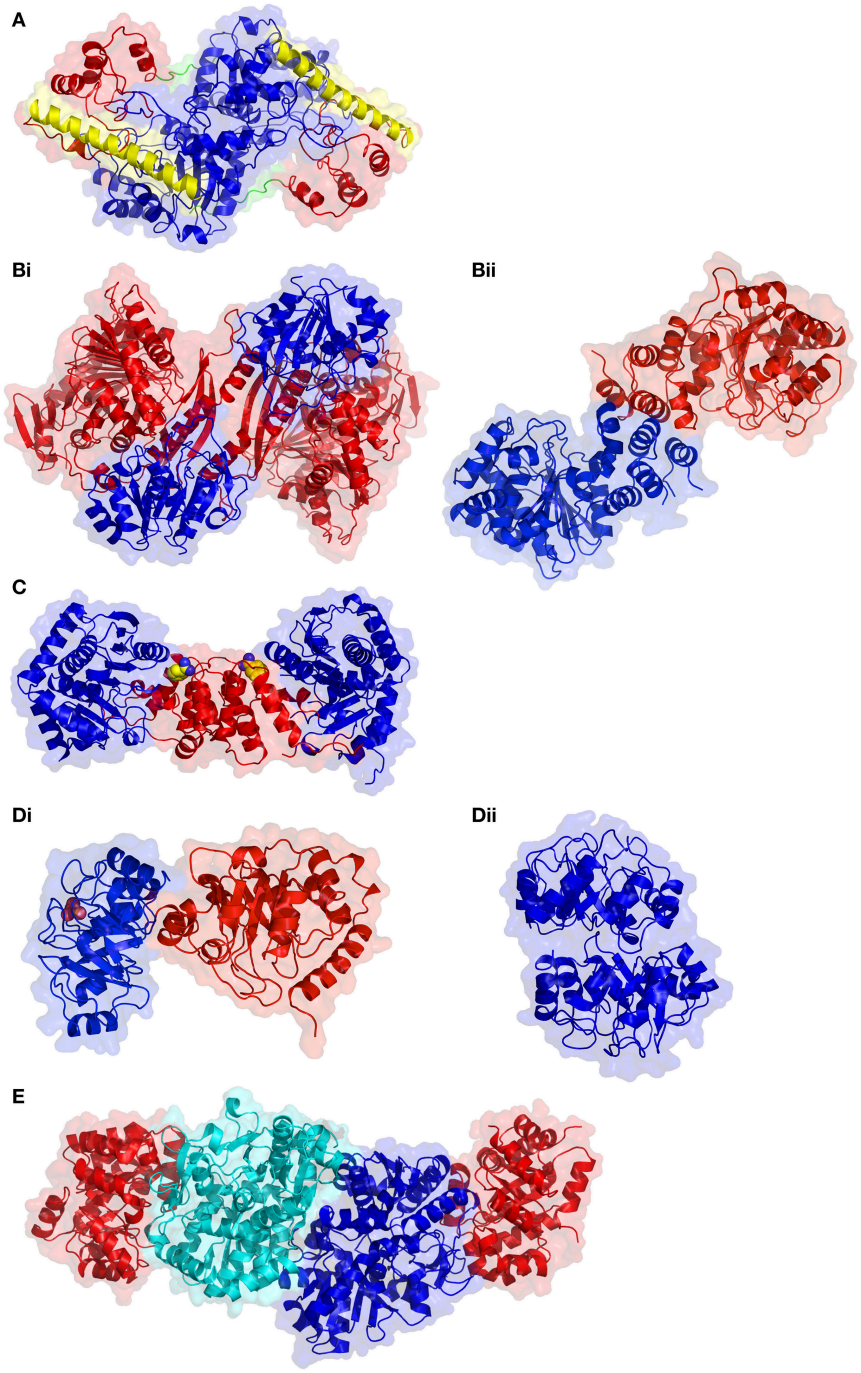
Structural comparison of enzymes involved in aromatic amino acid biosynthesis. **(A)** Tyrosine aminotransferase from *E. coli* (PDB code 3TAT). The large domain and small substrate binding domain are colored in blue and red, respectively. The α-helix that links the two domains is shown in yellow and the N-terminal arm in green. **(Bi)** Anthranilate synthase from *Serratia marcrescens* (PDB code 1I7S) in the α_2_β_2_ heterotetramer conformation. The α subunit is in blue and the β subunit in red. **(Bii)** Anthranilate synthase from *M. tuberculosis* in the homodimer conformation. **(C)** Anthranilate phosphoribosyltransferase from *M. tuberculosis* (PDB code 2BPQ). The two domains of each monomer, small and large, are colored red and blue, respectively, and the active site cleft is indicated by the bound benzamidine molecule, shown as spheres with carbon atoms colored yellow. **(Di)** Bifunctional *E. coli* phosphoribosyl anthranilate isomerase enzyme (colored in blue) and the indoleglycerolphosphate synthase domain colored red (PDB code 1PII). The phosphate, shown as pink spheres, identifies the position of the phosphoribosyl anthranilate isomerase enzyme active site. **(Dii)** Dimeric monofunctional phosphoribosyl anthranilate isomerase from *T. thermophilus* (PDB code 1V5X). **(E)** The heterotetrameric tryptophan synthase from *M. tuberculosis* (PDB code 5TCF). The α subunits are colored in red, while the β subunits are colored in blue and cyan to highlight the subunit interface.

Although the structure of a plant TAT remains unsolved, sequence analysis suggests that they likely assume the same configuration as the reported bacterial, mammalian and eukaryotic pathogenic examples. Structures of TAT show a homodimeric assembly, with each monomer having a small and large domain connected by a large α-helix (Figure 3A). The large domain contains αβα motifs folded into a central β-sheet that is flanked by α-helices on both faces. Two small β-strands and five α-helices contributed by the N- and C-terminal sequence regions of the enzyme interact to form the smaller domain. Distinct from the smaller domain is an extended N-terminal arm which extends toward and interacts with the large domain of the opposing monomer, imparting stability to the dimer. The active site is located between the two domains. A conformational change is induced by substrate binding, whereby the smaller domain shifts toward the larger domain closing the active site pocket.

### Tryptophan biosynthesis

**Figure 4:**
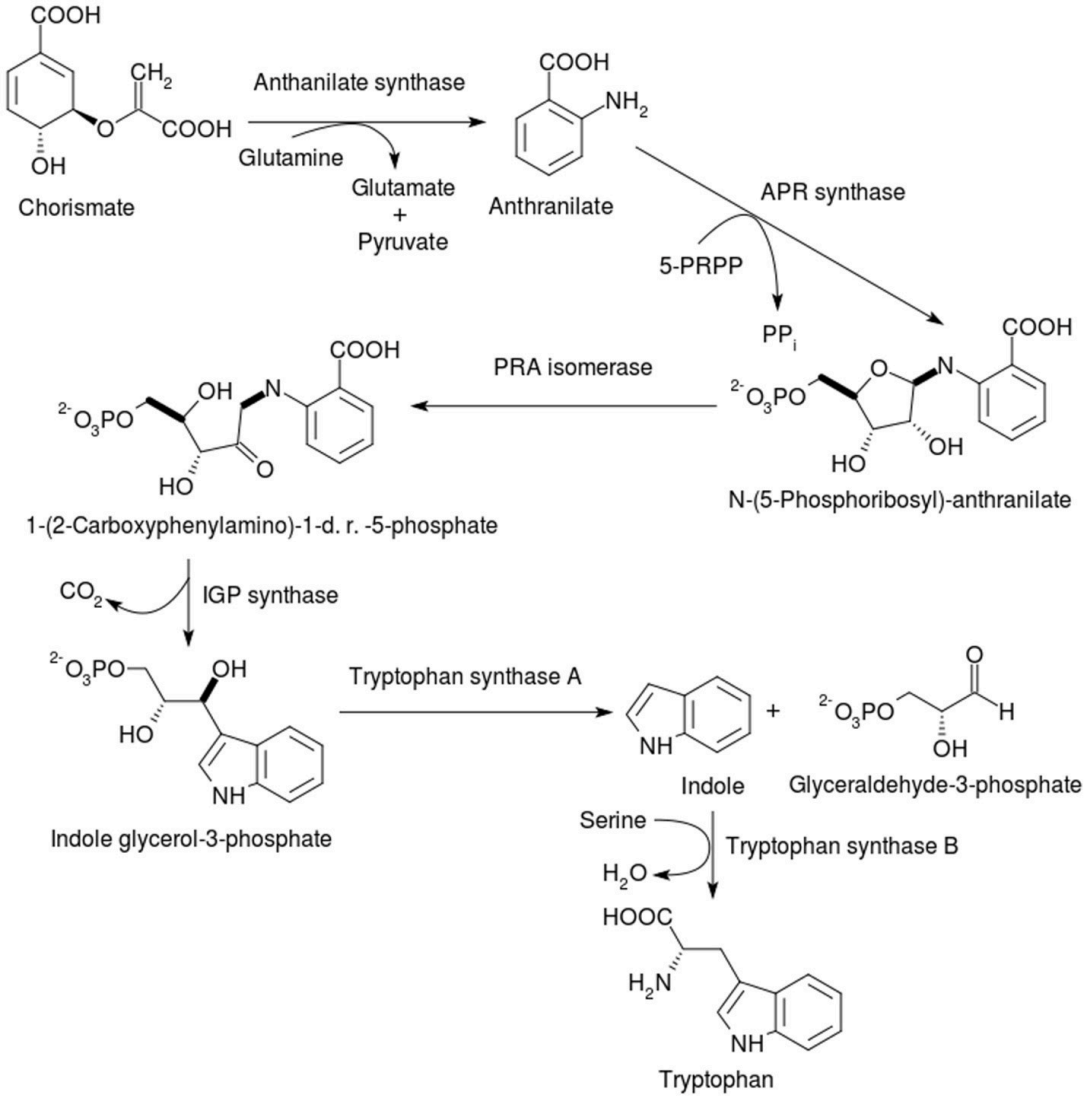
Synthesis of tryptophan from chorismate. After the seven-step pathway via shikimate generates chorismate, the biosynthesis of tryptophan diverges from those of the other two AAA. 5-PRPP = 5-Phosphoribose-1-diphosphate; APR synthase = Anthranilate phosphoribosyl synthase; PRA isomerase = Phosphoribosyl anthranilate isomerase; 1-(2-Carboxyphenylamino)-1-d. r.−5- phosphate = 1-(2-Carboxyphenylamino)-1-dexoyribulose-5- phosphate, IGP synthase = indole glycerol-4-phosphate. Tryptophan synthase A (or α-subunit) cleaves indole glycerol-3-phosphate into indole and glyceraldehyde-3-phosphate, while tryptophan synthase B (or β-subunit) is responsible for the PLP-dependent condensation of the latter two compounds into tryptophan.

Tryptophan is the most chemically complex and is the least abundant of the AAA. In animals, this “rare” amino acid must be obtained through dietary means or through sequestration from symbiotic organisms. The biosynthesis of tryptophan occurs only in plants and microbes, and therefore contains multiple attractive targets for the development of herbicides and antimicrobials. Chorismate is the branching point from where the biosynthesis of tryptophan diverges from that of phenylalanine and tyrosine. The pathway from chorismate to tryptophan is shown in Figure 4.

#### Anthranilate synthase (AS)

The enzyme catalyzing the committed step of tryptophan biosynthesis is anthranilate synthase (AS) (EC 4.1.3.27), which converts chorismate to anthranilate (Morollo and Eck, 2001; Tang et al., 2001; Lin et al., 2009). AS, also designated as TrpG or TrpE, belongs to the lyase family, in particular to the oxo-acid-lyases capable of cleaving carbon-carbon bonds. It is allosterically inhibited by the final product of the pathway, tryptophan, and is an important player in partitioning in chorismate toward tryptophan biosynthesis. The formation of anthranilate involves the transfer of ammonia from the donor glutamine to chorismate, generating glutamate and pyruvate (apart from anthranilate). This reaction is classified as a 1,4-nucleophilic substitution by ammonia followed by the elimination of pyruvate. Anthranilate synthase is considered to share evolutionary origins with other chorismate-metabolizing enzymes such as salicylate synthase (EC 4.2.99.21), aminodeoxychorismate synthase (ACDS) (EC 2.6.1.85) and isochorismate synthase (ICS) (EC 5.4.4.2), owing to similarities in reaction chemistry and polypeptide folding (Bulloch et al., 2004; He et al., 2004; He and Toney, 2006). It has been shown that *S. typhimurium* AS can accommodate water as a nucleophile (as in ICS) instead of ammonia, thereby displaying a bifunctional AS/ICS activity (Plach et al., 2015). Another study demonstrated that only a few mutations were sufficient to induce AS activity in an aminodeoxychorismate synthase, which in its native form does not eliminate pyruvate (Culbertson et al., 2015).

Anthranilate synthase contains two components, AS-I (TrpE), and AS-II (TrpG), (both EC 4.1.3.27). AS-I synthesizes the intermediate 2-amino-2-deoxyisochorismate (ADIC) from chorismate and ammonia (Morollo et al., 1993), while the amidotransferase activity of AS-II supplies the ammonia with glutamine as the donor (Mouilleron and Golinelli-Pimpaneau, 2007). The enzyme mechanism and active chemistry are described in detail in another review (Romero et al., 1995) and the references therein. Briefly, the AS-I subunits bind to aminate chorismate, when high concentrations of ammonia are present. The AS-II subunits releases ammonia for the amination of chorismate via the formation of a γ-glutamyl-*S*-cysteinyl enzyme intermediate. Magnesium is suggested to make the 4-hydroxyl group of chorismate a better leaving group.

Anthranilate synthase organization and size differs between bacteria and plants. Some bacterial AS contain the two subunits, α and β (Figure 3B), in various oligomeric compositions such as αβ, α_2_β_2_, or α_3_β_3_, or with a fusion of the two subunits (Romero et al., 1995; Ashenafi et al., 2015). Other bacterial AS such as the fused *Streptomyces venezuelae* are not only monomeric, but also cannot use ammonia instead of glutamine to aminate chorismate (Ashenafi et al., 2015). Among pathogenic bacteria from which crystallographic structures of AS are available, the oligomeric organization differs substantially between *Serratia marcrescens* and *S. typhimurium*, where the α_2_β_2_ tetramer associates via the AS-I subunits (Spraggon et al., 2001; Figure 3Bi), while in the *M. tuberculosis* enzyme, AS-I is a homodimer, even when AS-II is present (Bashiri et al., 2015; Figure 3Bii). In the last enzyme, the allosteric binding site for the inhibitor tryptophan is found near the interfacial region. The α-subunit of AS is encoded by the *TRP2* gene in fungi and *ASA1/ASA*2 in plants. In plants, AS contains only the *ASA* and *ASB1/ASB2/ASB3* gene products, usually with a α_2_β_2_ oligomeric state; the AS system is well-characterized in *A. thaliana* (Niyogi and Fink, 1992; Niyogi et al., 1993). It is noteworthy that in some eukaryotes such as fungi, the AS complex may contain enzyme subunits with other functions in the tryptophan biosynthesis, i.e., multi-functional subunits (Hütter et al., 1986). In plants, such as potato and tobacco, as well as in bacteria, such as *Pseudomonas putida*, the presence of tryptophan-sensitive and -insensitive AS isozymes have been suggested as indicative of the existence of complete but distinct pathways for both primary and secondary metabolism of tryptophan (Hrazdina and Jensen, 1992). It was reported that the second set of AS gene products in *P. putida* and *P. aeruginosa*, which are tryptophan-insensitive, participate in the biosynthesis of the blue-green phenazine pigment, pyocyanin (Essar et al., 1990).

#### Anthranilate phosphoribosyltransferase (AnPRT)

Also known as anthranilate phosphoribosyl synthase (APR synthase) or TrpD (EC 2.4.2.18), this enzyme catalyzes the transfer of a phosphoribosyl group from phosphoribosyl pyrophosphate (PRPP) to anthranilate, yielding phosphoribosylanthranilate (PRA) and eliminating pyrophosphate (2nd step in Figure 4). AnPRT is a member of the phosphoribosyl transferase (PRT) involved in nucleotide biosynthesis and salvage apart from AAA biosynthesis (Sinha and Smith, 2001). A number of microbial AnPRT have been studied in detail, with the *M. tuberculosis* enzyme showing some interesting structural features (Castell et al., 2013; Cookson et al., 2014, 2015). In this case, an unusual channel, which could deliver anthranilate to the active site, has been observed. Multiple anthranilate-binding sites have been reported within this channel and may account for the substrate inhibition caused by anthranilate. The biochemical role of this substrate channeling may be to deliver the PRPP to anthranilate (leading to phosphate attachment), instead of water (which would lead to hydrolysis). AnPRT inhibitors based on these multiple binding sites have been explored (Evans et al., 2014). AnPRT enzymes are homodimeric, with each subunit constructed from two domains that interact via a hinge region that contains the active site (Figure 3C). The smaller N-terminal domain is comprised of four α-helices while the larger α/β C-terminal domain is assembled by a central β-sheet made from seven β-strands, six parallel and one antiparallel, enveloped by eight α-helices.

#### Phosphoribosyl anthranilate isomerase (PRAI)

Also known as PRA isomerase or TrpF (EC 5.3.1.24), this enzyme is responsible for the isomerization of N-(5-phospho-beta-D-ribosyl)anthranilate (PRA) into 1-(2-carboxyphenylamino)-1-deoxy-D-ribulose 5-phosphate (CdRP) (3rd in Figure 4). The reaction involves an isomerization via an “Amadori rearrangement,” via a series of proton transfer steps leading to an enolamine, which then undergoes tautomerism to the ketoamine form (CdRP). The enzyme architecture varies widely, with some bacteria such as *E. coli* containing TrpF fused to the C-terminus of the next enzyme in the pathway (Wilmanns et al., 1992). Due to the existence of an analogous Amadori rearrangement in the biosynthesis of histidine catalyzed by the ProFAR isomerase (EC 5.3.1.16) (Henn-Sax et al., 2002), this enzyme (PRAI) has received attention from researchers seeking to understand the broader context of AAA biosynthesis and animal health. Indeed, a dual-functional enzyme called PriA has been discovered in *M. tuberculosis* and *S. coelicolor* (Due et al., 2011) and the structural basis for the bi-functionality has been explored in detail (List et al., 2011).

The bifunctional enzyme is monomeric with the C-terminal end facilitating the phosphoribosyl anthranilate isomerase reaction (Figure 3Di). In contrast, the mono-functional enzyme is monomeric in mesophiles, whereas in thermophiles where increased structural stability is required, the phosphoribosyl anthranilate isomerase is observed primarily as a dimer (Figure 3Dii). Regardless whether this reaction is catalyzed by a mono- or bi-functional enzyme, the domain responsible for the isomerization adopts the same basic (βα)_8_ fold. The active site is located at the C-terminal end of the barrel and in dimeric structures, the loops located at the N-terminal side of the barrel interlock the monomers together. An increased helical content and increased numbers of charged residues observed in the thermophiles has also been proposed to contribute to enzyme stability.

#### Indole glycerol phosphate synthase (IGP synthase)

TrpC or indole-3-glycerol-phosphate synthase (EC 4.1.1.48) catalyzes the penultimate step of tryptophan biosynthesis (Figure 4), which is the conversion of CdRP into indole-3-glycerol-phosphate (IGP). In some bacteria such as *E. coli*, the IGP synthase is fused with the previous enzyme in the pathway, leading to a bi-functional PRA synthase: IGP synthase (Wilmanns et al., 1992). In this enzyme, a conserved glutamate and two conserved lysine residues have been identified as essential for catalysis. A notable feature of the IGP synthase reaction is that it is a series of biochemical steps—condensation, decarboxylation and dehydration in that sequence, whose kinetic mechanisms have recently been elucidated (Schlee et al., 2013).

#### Tryptophan synthase

The final step of tryptophan biosynthesis (Figure 4) is catalyzed by tryptophan synthase or TrpAB (EC 4.2.1.20), which is one of the first enzymes known to catalyze two different reactions in two separate active sites connected to each other via a tunnel on the interior of the protein (Leopoldseder et al., 2006; Dunn, 2012). The enzyme consists of a α_2_β_2_ tetramer (Figure 3E), as demonstrated by the recent report of the *M. tuberculosis* crystal structure (Wellington et al., 2017). The α subunit generates indole from IGP by means of a retro-aldol type of reaction wherein glycerol-3-phosphate is eliminated, and channels the indole into the second active site, which is present in the β subunit. The second active site chemistry involves a typical PLP Schiff base mechanism. The activation of the substrate serine replaces an active site lysine attached to PLP. Indole acts as a nucleophile to displace a water molecule and the elimination-addition ends by indole condensing with a 3-carbon unit to form tryptophan. The shielding of reaction intermediates from the bulk solution by tunneling between the active sites and the complex allosteric coupling of the bound subunits have been the subject of many structural and mutagenesis studies. NMR studies, in particular, have shed light on the intricate dance of the tryptophan synthase components (Axe and Boehr, 2013) and an extensive account of the workings of tryptophan synthase nanomachine is available elsewhere (Dunn et al., 2008).

### AAA biosynthesis in pathogenic microbes and chemotherapeutic interventions in animal health

Since animals lack the AAA biosynthetic pathways, pathogens that biosynthesize AAA are attractive targets for developing new anti-infective substances, especially since the rise of antibiotic resistance threatens the effectiveness of traditional antimicrobials. An increase in the number of available structures of enzymes in protein structure databases solved by X-ray crystallography (particularly enzymes from pathogenic microbes), combined with recent improvements in computational methods applied to elucidate and understand enzyme function as well as the increasing pace of genome sequencing and annotation of microbes in the last decade, have opened up enormous opportunities to develop new antimicrobial targets as well as to discover new antimicrobial molecules. The shikimate pathway furnishes not only the AAA, but also molecules such as vitamins and cofactors, and therefore is attractive for the discovery and or development of new chemotherapeutic agents (Lamichhane et al., 2011).

Enzymes involved in the shikimate pathway have been detected in the protists, *Toxoplasma gondii* (which causes toxoplasmosis) and one of the malarial parasites, *Plasmodium falciparum* (Roberts et al., 1998; Campbell et al., 2004). However, only the last enzyme of the pathway, chorismate synthase has been identified conclusively on the basis of genome annotation, and the remaining enzymes seem to be missing. It is possible that the functions of the “missing enzymes” are performed by others which are not homologs of well-known examples, or by homologs which have diverged significantly from known examples, so that they are not identified easily (McConkey et al., 2004). Six of the seven enzymes of the pathway were identified in *M*. *tuberculosis* (Cole et al., 1998), the *aroF, aroG, aroB, aroD, aroE, aroK* and *aroA* genes, are considered to be essential for survival of this pathogen (http://www.essentialgene.org/ Database of essential genes)^1^. For *Helicobacter pylori*, the causative agent of gastric ulcers and a type I carcinogen, four of the seven shikimate pathway genes, *aroQ, aroE, aroK*, and *aroC*, are essential. It can be seen that SK (*aroK*) and type II DHQ (*aroD/aroQ*) are important in both tuberculosis and *H. pylori* infections, and therapeutics based on inhibiting these two enzymes were reviewed extensively elsewhere (González-Bello, 2016).

#### Shikimate kinase (SK) inhibitors

SK phosphorylates shikimate at the 3-hydroxy group, at the expense of ATP. *E. coli* contains two types of enzymes SK1 (AroK) and SK II (AroL), but most bacteria have only one SK variant. SK inhibitors have been developed both by a substrate-mimetic strategy as well as by screening compound libraries. The P-loop is conserved in many ATP- and GTP-dependent proteins. Therefore, substrate mimics of shikimic acid were designed to bind the substrate-binding (SB) site of SK specifically (Blanco et al., 2013). Many of the compounds developed in that study for *M. tuberculosis* SK (Mt-SK) were reversible competitive inhibitors and some, such as the 3-aminoshikimates closely resembled the structure of shikimate. The inhibition kinetics and molecular modeling of these compounds showed that fixing the C4 and C5 hydroxyl groups in the diaxial conformation in the substrate mimic might be a good way to inhibit SK, due to the dramatic reduction in the flexibility of two domains—(1) the SB domain (residues 9–17 in Mt-SK) and (2) the LID domain (residues 112–124 in Mt-SK). Arg 116 was identified as a key residue during ATP-binding, product release and also a Lewis acid during catalysis. Other SK-targeting compounds have been discovered, for example, an LC-MS based screening of around 400 compounds at the National Institutes of Health (NIH) Tuberculosis Antimicrobial Acquisition and Coordination Facility identified three inhibitors with sub-micromolar IC_50_ values for Mt-SK (Simithy et al., 2014) and a high throughput screening effort led to the discovery of inhibitors of *H. pylori* SK (Han et al., 2007). High-throughput virtual screening efforts have also been reported (Segura-Cabrera and Rodríguez-Pérez, 2008; Coracini and de Azevedo, 2014).

#### Dehydroquinase (DHQ) inhibitors

DHQ catalyzes the dehydration of 3-dehydroquinate to 3-dehydroshikimate, a reversible reaction. Two forms of DHQ with no similarity at the level of primary sequences, and differing biochemical and biophysical properties are found (Kleanthous et al., 1992; Gourley et al., 1999). DHQ1 (AroD) found in plants, fungi and some bacteria such as *E. coli* and *Salmonella typhi*, catalyzes a *syn* dehydration based on a Schiff base mechanism. Apart from biosynthesis, DHQ1 may also be required for virulence in some bacteria (Racz et al., 2013). DHQ2 (AroD/AroQ) catalysis on the other hand, does not entail the formation of a covalent Schiff base intermediate. It is essential in prominent pathogenic species such as *M. tuberculosis* and *H. pylori* (Database for essential genes). In Mt-DHQ2 (*M. tuberculosis* DHQ2), a conserved aspartate (Asp 88) residue from an adjacent enzyme subunit triggers the conversion of an essential tyrosine (Tyr24) into tyrosinate, while the positive charge of Arg19 stabilizes the enolate intermediate generated from dehydroquinate.

The reaction was suggested to go through an enolate instead of an enol intermediate, due to the significantly lower energy of the enol intermediate (Blomberg et al., 2009); many reversible competitive inhibitors of Mt-DHQ2 were developed based on this proposed mechanism (González-Bello and Castedo, 2007; Yao and Li, 2014). Since the formation of the enolate involves abstraction of the C2 axial hydrogen by Tyr24, replacement of this hydrogen by another group is expected to inhibit the enzyme. However, substitution of the C2 equatorial hydrogen by benzyl groups also generates effective inhibitors as the methylene group permits close stacking of the benzene ring close to the aromatic ring of the Tyr 24 residue (González-Bello et al., 2008; Prazeres et al., 2009). The reaction mechanism also entails a ring-flattening between the C2 and C3 positions during the elimination. Therefore, compounds mimicking the enolate transition state could be good competitive inhibitors and the first inhibitor synthesized and tested using this approach was 2,3-dehydroquinic acid (Frederickson et al., 1999). It was shown to be a reversible competitive inhibitor of Mt-DHQ2 and Sc-DHQ2 (*S. coelicolor*-DHQ2). The solution of the X-ray diffraction structure of Sc-DHQ2 with an inhibitor containing a C2-C3 double bond bound at the active site (PDB entry 1GU1, 1.8 Å, Roszak et al., 2002) spurred the development of improved inhibitors. An important feature of DHQ2 substrate recognition is the carboxylate binding pocket which has been a challenge for *in vitro* optimization of anti-tuberculosis drugs, but an ester prodrug approach was demonstrated to improve antibacterial activity by increasing the cell permeability of mycobacteria (Tizón et al., 2011). Several drug-like aromatic Mt-DHQ2 inhibitors with anti-tubercular activity in the micromolar range have been described, including some nitrobenzyl-gallate analogs (González-Bello et al., 2009; Schmidt et al., 2013).

#### Tryptophan biosynthesis inhibitors with a focus on tuberculosis (TB)

The emergence of multi-drug resistant (MDR) and extreme drug resistant (XDR) TB with cure rates of around 50 and 26% respectively has rendered many TB treatments ineffective (Ma et al., 2010), and represents a significant increase in the associated mortality rate. TB is one of the great public health challenges and the need for new drugs, especially those with novel targets and modes of action (Ma et al., 2010). This has generated interest in the metabolism of the TB pathogen. Although the complete genome sequence of *Mycobacterium tuberculosis* has been published (Cole et al., 1998), an unambiguous identification of all the genes encoding enzymes in this pathway has not been accomplished. The genes already identified in this context are also not arranged in a single open reading frame (ORF) and were therefore identified only after genetic complementation and biochemical studies. The work of the TB Structural Genomics Consortium (TBSGC) enabled the determination of crystal structures of many proteins from *M. tuberculosis* including those involved in tryptophan metabolism such as, TrpB, TrpC, and TrpE (Lee et al., 2006; Czekster et al., 2009; Shen et al., 2009b). TrpD was shown to be necessary for colonization of the lungs (Lee et al., 2006).

Remarkably, a tryptophan auxotrophic strain was not virulent even in mice with severe combined immunodeficiency (Smith et al., 2001), showing that tryptophan biosynthesis is critical for initiating and sustaining TB infections. A decrease in tryptophan concentration and an increase in the activity of indoleamine-2,3-dioxygenase (IDO) (EC 1.13.11.52), an enzyme from the kynurenine pathway (please see section AAA decarboxylase (AADC) structure and pathology), was observed in pulmonary TB cases, both in the serum (Suzuki et al., 2012) and the pleural fluid of the lungs (Suzuki et al., 2013). Therefore, it could be inferred that tryptophan degradation was induced specifically in the lungs by the host as a tryptophan starvation response to counter the pathogen. While plant tryptophan synthase inhibitors with sub-micromolar potency (Sachpatzidis et al., 1999) were predicted to be useful against TB, none have been tested as TrpAB inhibitors in the pathogen (Dias et al., 2006). A sub-micromolar inhibitor for TrpC was reported (Shen et al., 2009b), whereas many inhibitors for TrpE are known. Curiously, inhibitors designed with one enzyme in the biosynthetic pathway as targets, often have activities against other enzymes in the pathway (Kozlowski et al., 1995). One of the two putative anthranilate synthase genes in TB was found to be a salicylate synthase involved in mycobactin synthesis (Harrison et al., 2006).

A transposon mutagenesis approach facilitated the discovery that an intact tryptophan biosynthetic pathway was necessary for pathogen survival in immunocompetent mice, but not those lacking CD4^+^ T cells (Zhang et al., 2013). The same authors showed further that TrpE knockout mutants of the bacterium were killed both *in vitro* and inside macrophages. Novel IGP synthase inhibitors that are effective against MDR TB have been reported (Shen et al., 2009a). Anthranilates fluorinated at the 5- or 6- position of the benzene ring were demonstrated to be antibacterial, with modest *in vivo* activity in mouse models (Zhang et al., 2013). Since the fluoro-anthranilates were not shown to be TrpD inhibitors for the *E. coli* enzyme, but alternate substrates leading to formation of fluorinated IGP *in vitro* (Cookson et al., 2014), the anti-tubercular activity of fluoro-anthranilates might stem from two possibilities. Either, the biosynthesis is inhibited downstream of TrpD, or the fluorinated tryptophan formed at the end of this pathway is toxic to the bacterium. Incorporation of fluorine at the 4-, 5-, or 6- positions of tryptophan was shown to be toxic to *E. coli*, possibly due to detrimental effects on protein structure (Brown et al., 1970).

### AAA catabolism in plants

#### Complete AAA degradation

Complete phenylalanine degradation has not been reported in plants (Mazelis, 1980) and phenylalanine hydroxylase homologs are absent in plants as far as the published data indicate, with no candidates found in the genome of the model plant *Arabidopsis thaliana*. However, a unique phenylalanine hydroxylase dependent on folate was discovered in non-flowering plants, which is localized in the chloroplast (Pribat et al., 2010). Complete degradation of tyrosine in *A. thaliana* was shown to proceed by the same pathway as in mammals (Dixon and Edwards, 2006). The transamination of tyrosine by TAT occurs yielding 4-hydroxyphenylpyruvate, which is then transformed by 4-hydroxyphenylpyruvate dioxygenase to homogentisate. Homogentisate in plants acts as the precursor for tocopherols such as vitamin E and plastoquinones. Further catabolic steps convert homogentisate via ring cleavage ultimately into fumarylacetoacetate. Hydrolysis of fumarylacetoacetate yields fumarate and acetoacetate, thereby linking tyrosine and fumarate metabolism, potentially both inside and outside of mitochondria. Although tryptophan has been shown to be a precursor for the synthesis of many secondary metabolites such as auxins, phytoalexins, glucosinolates, and alkaloids (Radwanski and Last, 1995), to date, the elucidation of the tryptophan degradation pathway(s) has not been reported in plants.

#### Partial degradation of phenylalanine

In plants, phenylalanine and tyrosine are catabolized to generate anabolic precursors for the phenylpropanoid pathway. Phenylalanine can be converted to cinnamate by the enzyme phenylalanine ammonia lyase (PAL) (EC 4.3.1.24). The expression of PAL-encoding genes are highly regulated by different biotic and abiotic stresses, and conditions which increases the requirement of the cell wall component lignin (Anterola and Lewis, 2002). Cinnamate can be further metabolized to *p*-coumaroyl CoA, a central metabolite in the phenylpropanoid pathway, which are involved in mediating responses pertaining to biotic and abiotic stresses (Dixon, 2001; Casati and Walbot, 2005). The phenylpropanoid pathway has been reviewed extensively elsewhere (Boudet, 2007; Vogt, 2010). These compounds impart mechanical strength to plant cells, and also participate in pest deterrence, drought resistance, UV protection, disease resistance, pollen viability and so on (Nair et al., 2004). Compounds such as lignans, lignins, cutin, suberin, catechins, sporopolleins, flavonoids, isoflavonoids, proanthocyanidins, aurones, phenylpropenes, stilbenes, alkaloids, and acylated polyamines are derived from this pathway and involved in plant defense. In addition, some of these compounds are involved in the synthesis of colorful pigments that are present in flowers and fruits (Fraser and Chapple, 2011).

Newer genome-based approaches, such as the creation of an extensive database for P450 superfamily genes (CYPedia) based on the microarray analysis of *A. thaliana* and the analysis of over 4,100 re-annotated genes predicted to be active in plant metabolism for co-expression with P450 genes, have been described recently (Ehlting et al., 2008) and have enabled the discovery of organ-specific expression of phenylalanine catabolic pathways in stamen (Alves-Ferreira et al., 2007), flower buds (Fellenberg et al., 2009), and pollen (Matsuno et al., 2009). Phenylalanine-derived volatile compounds are involved in plant reproduction and defense (Dudareva et al., 2006; Schaller, 2008). Phenylpropanoids, benzenoids, phenylpropenes, and nitrogenous aromatics are the major classes of volatiles in this context. Phenylalanine is converted to phenylacetaldehyde by oxidative decarboxylation (Kaminaga et al., 2006). Apart from phenylacetaldehyde, other phenylalanine-based volatiles include phenylethylacetate, 2-phenylethanol, methylbenzoate, and isoeugenol (Watanabe et al., 2002; Verdonk et al., 2003; Baldwin et al., 2004; Schuurink et al., 2006; Tieman et al., 2006; Ben Zvi et al., 2008; Gonda et al., 2010; Klee, 2010). Phenylalanine is also the precursor for a class of sulfur-containing secondary metabolites called phenylalanine glucosinolates (Reichelt et al., 2002).

#### Partial degradation of tyrosine

Tyrosine, instead of phenylalanine, is the direct precursor of coumarate in the phenylpropanoid pathway in some plants (Neish, 1961; MacDonald and D'Cunha, 2007), where the enzyme responsible for the transformation is tyrosine-ammonia-lyase (TAL) (EC 4.3.1). Tyrosine decarboxylase (TyrDC) (EC 4.1.1.25) is a PLP-dependent enzyme that removes CO_2_ from tyrosine to produce tyramine. TyrDC is distributed across the plant kingdom and is involved in the biosynthesis of defense compounds such as glycosides (Ellis, 1983) and alkaloids (Leete and Marion, 1953). The induction of TyrDC was shown to be induced upon wounding or fungal elicitor treatment (Kawalleck et al., 1993; Trezzini et al., 1993; Guillet and De Luca, 2005). In addition, recent studies using *A. thaliana* have demonstrated that TyrDC is involved in abiotic stress response especially during drought and exposure to high salt concentrations (Lehmann and Pollmann, 2009). TyrDC in *A. thaliana* also feeds into the production of alkaloids as well as cell-wall hydroxycinnamic acid amides (Facchini et al., 2000). Tyrosine catabolism also leads to the synthesis of the isoquinoline alkaloids, which are a major class of secondary metabolites found in at least 20% of all plant species (Facchini et al., 2004).

Tyrosine also serves as the starting compound for the biosynthesis of tocochromanols (DellaPenna and Pogson, 2006; Mène-Saffranè and Dellapenna, 2009) as well as plastoquinones (Norris et al., 1995), with the former class being essential antioxidants (Vitamin E) in the diets of animals (Schneider, 2005). The committed step of tocochromanol biosynthesis involves TAT, which converts tyrosine into *p*-hydroxyphenylppyruvate (Norris et al., 1995; Garcia et al., 1999; Lopukhina et al., 2001). Tyrosine is the precursor for *meta*-tyrosine, a non-proteogenic amino acid found in fescue grasses. It has been hypothesized that *meta*-tyrosine can be incorporated into proteins instead of phenylalanine by eukaryotic phenylalanine-tRNA synthases (Duchêne et al., 2005; Klipcan et al., 2009). The incorporation of *meta*-tyrosine can lead to wide range of plant growth defects including growth retardation and inhibition of root development (Bertin et al., 2007).

#### Partial degradation of tryptophan

Tryptophan is the precursor to the family of auxins hormones (Gibson et al., 1972; Wright et al., 1991; Radwanski and Last, 1995; Tsurusaki et al., 1997; Ostin et al., 1998). While indole-3-acetic acid (IAA) is the most abundant auxin, other indole-containing auxins such as; 4-chloro-indole-3-acetic acid (4-Cl-IAA), indole butyric acid (IBA) and indole propionic acid (IPA) are also important and have integral roles in plants. IAA, henceforth simply “auxin” in this article, is essential to almost all of the major developmental processes in plants including embryogenesis, seedling growth, root elongation, vascular patterning, gravitropism, and flower development (Davies, 2004). Asymmetric auxin distribution in response to environmental cues govern the form, shape, strength and direction of growth of all organs and the interactions between various organs (Benkov et al., 2003). At least four pathways have been proposed for the production of IAA from tryptophan. It should be noted that the complete pathways for the degradation of IAA are still not elucidated (Strader and Bartel, 2008).

The two-step auxin biosynthesis pathway via indole-3-pyruvate (IPy), which is highly conserved throughout the plant kingdom and has been characterized in several monocot and dicot plants, is well known. The first step of this pathway is the elimination of the amino group from the AA by the tryptophan aminotransferase (TAA) (EC 2.6.1.1) family of transaminases to generate IPy. The latter compound then undergoes oxidative decarboxylation catalyzed by the YUC family of flavin monooxygenases to produce IAA. The enzymes of the transaminase-dependent pathway for IAA biosynthesis were characterized *in vitro* in the recombinant enzyme (Stepanova et al., 2008; Tao et al., 2008). Recombinant TAA1 catalyzes the PLP-dependent transfer of an amino group from tryptophan to 2-oxoglutarate, yielding IPy and glutamate. Disruption of TAA genes not only abolishes IPA production, but also affects the metabolism of other α-ketoacids and amino acids. Recombinant YUC6 from *A. thaliana* was purified and shown to be a FAD-containing enzyme, wherein NADH reduces the bound FAD to FADH_2_, which then reacts with molecular oxygen to form the C4α-(hydro) peroxyflavin intermediate that is the actual oxidizing species (Dai et al., 2013).

Other routes that generate IAA from tryptophan include (1) the indole-3-acetaldoxime (IAOx) pathway, which contains the two cytochrome P450 enzymes CYP79B2 and CYP79B3 (EC 1.14.13) (Hull et al., 2000; Bartel et al., 2001), (2) the indoleacetamide pathway, which involves the same two P450 enzymes during the initial steps (Pollmann et al., 2002), and (3) the tryptamine (YUCCA) pathway that involves catalysis by tryptophan decarboxylase (TDC) (EC 4.1.1.28) (Takahashi, 1986; Facchini et al., 2000; Quittenden et al., 2009). In addition, a putative tryptophan-independent pathway of IAA biosynthesis directly from indole has been proposed (Normanly et al., 1993; Radwanski et al., 1996).

Enzymatic decarboxylation of tryptophan by the PLP-dependent TDC produces the indole alkaloid tryptamine, which is found in small amounts in many plants. It is deemed to be a feedstock compound for pathways involved in synthesis of terpenoid indole alkaloids (TIA) and those that influence growth and the microbiome. Expression of TDC and TYDC in transgenic tobacco depleted the pools of tryptophan and tyrosine respectively, but in addition also perturbed pathways not directly involving AAA, such as methionine, valine, and leucine biosynthesis (Guillet et al., 2000). Tryptophan is also converted to compounds associated with plant-insect and plant-pathogen interactions known as the indole glucosinolates (Halkier, 1999), which are natural products containing thioglucose and sulfonate bound to the oxime derived from of the amino acid bound to an oxime function (Halkier and Gershenzon, 2006). IAOx also feeds into the indole glucosinolate pathway via an oxime-metabolizing enzyme CYP83B1 (Naur et al., 2003). Another major category of tryptophan-derived secondary metabolites are the phytoalexins (Pedras et al., 2000). The major indolic phytoalexin is camalexin, which accumulates upon infection with pathogens or the action of abiotic elicitors (Zhao and Last, 1996; Böttcher et al., 2009).

### AAA biosynthesis and catabolism in plant health

In plants, chorismate is not only a precursor of the three AAA, but also the initial compound for the biosynthesis of folates, such as tetrahydrofolate or vitamin B9 (Basset et al., 2004; Waller et al., 2010), pigments (Gross et al., 2006; Kim H. U. et al., 2008) and isochorismate en route to salicylate (Wildermuth et al., 2001; Garcion et al., 2008). Therefore, the shikimate pathway could potentially be engineered to augment the synthesis of folates or vitamin K in crop plants. Another enzyme in the shikimate pathway, 5-enolpyruvylshikimate-3-phosphate synthase (EPSPS), is the target of the well-known herbicide, *N*-phosphonomethylglycine or glyphosate commonly referred to as Roundup®, which is a mimic of PEP and competitively inhibits EPSPS, thereby reducing the carbon flux through the pathway (Healy-Fried et al., 2007). Non-plant EPSPS are used to provide herbicide resistance in transgenic crops (Duke and Powles, 2008). Being the basis for Roundup-Ready transgenic crops, EPSPS has received much research attention (Singer and McDaniel, 1985; Smart et al., 1985; Duke and Powles, 2008).

The biosynthesis of AAA and secondary metabolites derived from them are often elevated in infection responses (Ferrari et al., 2007). Manipulation of these responses could help improve plant protection against bacterial disease. Invading bacteria trigger the transcription of pathways including AAA metabolism and pigment biosynthesis within 12 h of infection (Truman et al., 2006). Salicylic acid (SA) is a plant defense compound that accumulates in leaves in response to local and systemic acquired resistance against phytopathogens (Malamy et al., 1990; Métraux et al., 1990; Ryals et al., 1996; Dorey et al., 1997; Dempsey et al., 1999). SA applied externally on plant surfaces alone is able to trigger enhanced resistance to pathogens in *A. thaliana*. Although, SA was initially shown to be synthesized from phenylalanine via the PAL pathway, inhibition of this pathway did not prevent the synthesis of SA (Mauch-Mani and Slusarenko, 1996; Coquoz et al., 1998), suggesting the existence of additional anabolic pathways. It was shown in further studies that chorismate was converted into isochorismate by isochorismate synthase (ICS) (EC 5.4.4.2), followed by the cleavage of isochorismate into SA and pyruvate; SA made by this pathway was necessary for local and systemic acquired resistance (Wildermuth et al., 2001).

Tryptamine production in transgenic tobacco was shown to severely inhibit the reproduction of whiteflies (Thomas et al., 1995), suggesting that tryptophan decarboxylase (TDC) is induced in response to pest attack in some plants. Since this work was done with transgenic plants, the possibility exists for a generic TDC-based plant protection strategy against whiteflies. The role of *meta*-tyrosine in inhibiting the growth of competing plants by fescue grasses (Bertin et al., 2007) has already been mentioned. Tryptophan is the precursor of serotonin, which has multiple functions in plants. Tryptophan is decarboxylated to tryptamine by TDC, which then undergoes hydroxylation by a cytochrome P450 monooxygenase, forming serotonin (Schröder et al., 1999). In dry seeds, serotonin is a sink for ammonia which can be toxic. Serotonin is present in plant spines, such as those of stinging nettles and the pain caused as a result of contact with them (Chen and Larivier, 2010), may deter browsing animals from consuming the plants. Since serotonin also affects the gut of animals, plants produce it in seeds and fruits as a way to promote the passage of seeds through the animal digestive tract in order to aid seed dispersal (Feldman and Lee, 1985). Serotonin is further metabolized into the growth regulator melatonin, which is also synthesized in response to various biotic and abiotic stresses, such as pathogenic fungi, toxins, soil salinity, drought and extreme temperature (Arnao and Hernández-Ruiz, 2015). Tryptophan is a precursor of thioquinolobactin, an antifungal agent that protects plants against the pathogen *Pythium debaryanum* (Matthijs et al., 2007) and is synthesized via a unique pathway involving xanthurenic acid (an intermediate of the kynurenine pathway) and a sulfurylase enzyme (Matthijs et al., 2004; Godert et al., 2007).

### AAA catabolism in animals

AAA obtained by animals from the diet can be broken down or converted into other necessary compounds, such as neurotransmitters (see Figure 1). Phenylalanine is often converted into tyrosine in animals and both these AAA feed into the biosynthesis of neurotransmitters, such as L-3,4-dihydroxyphenylalanine (L-DOPA), dopamine, epinephrine, and norepinephrine (Figure 1). Tryptophan is a precursor for the synthesis of neurotransmitters such as; serotonin and tryptamine, the neurohormone melatonin, the vitamin niacin, the enzyme cofactors NAD^+^ and NADP^+^, in addition to the neuroprotectant kynurenine. It is not surprising, therefore, that metabolic defects in animals genes related to AAA catabolism have significant effects on their health. We will limit this review to a description of key AAA catabolic pathways in animals, along with a brief general discussion of pathologies related to each AAA catabolic pathway.

Complete AAA degradation pathways described for plants also occur in animals, whereby they break down phenylalanine and tyrosine from proteins for recycling. Alkaptonuria is an inherited disorder affecting this function, caused by non-functional and or suboptimal activity of the enzyme homogentisate 1,2-dioxygenase dioxygenase (HGD) (EC 1.13.11.5) (Zatkova, 2011). Tyrosinemia is an inherited disorder in a single pathway involving mutations in one of three distinct enzymes involved in tyrosine degradation—fumaroylacetoacetate hydrolase (EC 3.7.1.2) (Grompe et al., 1994), TAT (James et al., 2005) or 4-hydroxyphenylpyruvate dioxygenase (Hpd) (EC 1.13.11.27), the last of which is very rare.

#### The monoamine and trace amine pathways

This is a major pathway for the catabolism of phenylalanine and tyrosine catabolism in animals. Here, many enzymes have additional roles in the synthesis of multiple neuroactive substances. The trace amines include all the neurotransmitters and neuroactive intermediates in this pathway except for L-DOPA, dopamine, epinephrine (adrenaline) and norepinephrin (noradrenaline). It enables the biosynthesis of the neurotransmitters phenylethylamine and *N*-methylphenylethylamine directly from phenylalanine, in addition to dopamine, octapamine, tyramine, *N*-methyltyramine, syneprhine, 3-methoxytyramine, epinephrine and norepinephrine either directly from tyrosine or from phenylalanine, which is hydroxylated to tyrosine. The physiological effects of these monoamine neurotransmitters are reviewed elsewhere (Broadley, 2010).

##### AAA hydroxylases (AAAH)

Phenylalanine is converted into tyrosine by phenylalanine 4-hydroxylase (PheOH or PAH) (EC 1.14.16.1), a member of the biopterin-dependent aromatic amino acid hydroxylases (AAAH) family, whose other members are tyrosine 3-hydroxylase (TyrOH) (EC 1.14.16.2), and tryptophan 5-hydroxylase (TrpOH or TPH) (EC 1.14.16.4). Tyrosine hydroxylase converts tyrosine to L-DOPA, which is rate limiting for the synthesis of the catecholamines dopamine, epinephrine and norepinephrine. Tryptophan hydroxylase converts tryptophan into 5-hydroxy-L-tryptophan en route to serotonin. All the AAAH enzymes contain iron and catalyze AAA hydroxylation using tetrahydrobiopterin. They act as rate-limiting enzymes in their respective pathways (Grenett et al., 1987). Detailed reviews of AAAH structural biology (Flatmark and Stevens, 1999), regulation (Fitzpatrick, 2015) and AAAH-based therapeutic targets (Waløen et al., 2016) have been published.

PheOH deficiency causes phenylketonuria (PKU) in humans, which is an inborn error of metabolism attributed to a single gene defect (Erlandsen et al., 1997). PKU leads to a deficiency of tyrosine, which is continuously produced from phenylalanine in many animals for the synthesis of the catecholamine and trace amine neurotransmitters. Untreated PKU can lead to seizures, intellectual disability, behavioral problems, and mental disorders (Al Hafid and Christodoulou, 2015). Most of the more than 300 mutations in this enzyme (PAH DB)^2^ are linked to PKU, while a few different mutations have been identified among patients suffering from non-PKU hyperphenylalaninemia (HPA). 36 PKU-linked mutations have been studied using *in vitro* expression systems, with the mutation sites mapping to the full length PheOH structure (Nowacki et al., 1998). In humans, knockout mutations in PheOH are not lethal, but the loss of TyrOH is, with the victims dying at a late embronic stage or briefly after birth (Flatmark et al., 1997). Only two TyrOH mutations were so far associated with disorders of the basal ganglia (Knappskog et al., 1994; Lüdecke et al., 1996). In later studies, the human TyrOH locus has also been linked to bipolar disorder (Smyth et al., 1997) and schizophrenia (Thibaut et al., 1997).

Humans have two distinct of the TPH gene, with an overall sequence identity of 71% (McKinney et al., 2005). Even though their biochemical reaction mechanisms are the same and their substrate specificities are similar, they have different expression and regulation patterns, as well as different physiological roles (McKinney et al., 2005). TPH1 synthesizes most of the serotonin in circulation and is expressed chiefly in the gastrointestinal tract, adrenal glands, kidneys, and the pineal gland. TPH2 however, occurs in the serotonergic neurons with wide distribution in various cortices in the brain (Amireault et al., 2013).

##### The structures of phenylalanine hydroxylase (PheOH) and tyrosine hydroxylase (TyrOH)

Each AAAH contains a non-heme iron center and a 6(*R*)-L-erythro-5,6,7,8-tetrahydrobiopterin (BH4) cofactor, and requires a dioxygen molecule during catalysis. The cofactor is oxidized to quinonoid dihydrobiopterin (qBH2), which is regenerated to BH4 by the NAD(P)H-dependent dihydropteridin reductase. The AAAH are all homotetramers, with each subunit consisting of a catalytic domain which has high homology (sequence identity over 80%) and a regulatory domain which is divergent (Hufton et al., 1995).

The structure of human PheOH (hPheOH) has been solved and shows an active site which is very open to the solvent and to the binding of exogenous ligands (Kappock and Caradonna, 1996; Fusetti et al., 1998). The negative potential and hydrophobic nature of the active site is considered to promote the binding of positively charged amphipathic molecules such as the actual substrates, pterin cofactors, and inhibitors such as catecholamines (Hufton et al., 1995). The catalytic iron is situated at the entrance of the pocket containing the active site, with space enough for both the pterin cofactor and the substrate (Hufton et al., 1995). A highly conserved motif 27 amino acids long has been proposed to govern the binding of the cofactor tetrahydrobiopterin (Jennings et al., 1991; Hufton et al., 1995). The competitive inhibition of PheOH and TyrOH by catecholamines has been investigated using binary complexes of the dimeric proteins with various catecholamines. The molecular basis for the inhibition has been proposed to be the binding of the inhibitors directly to the catalytic iron center via the bidentate coordination of the two hydroxyl groups (Erlandsen et al., 1998).

The regulatory domains have also been the subject of structural studies; the solution structure of the regulatory domain of TyrOH shows a core ACT domain similar to that found in PheOH. When isolated, this domain of TyrOH forms a stable dimer, whereas the corresponding domain in PheOH exhibits an equilibrium between the monomer and dimer, with dimer stabilization afforded by the substrate phenylalanine. This correlates well with the fact that TyrOH is regulated by the binding of catecholamines, while PheOH is regulated by the substrate binding to an allosteric site (Fitzpatrick, 2015).

##### AAA decarboxylase (AADC) and other enzymes

Phenylalanine is converted to the neurotransmitter phenylethylamine by the PLP-dependent enzyme aromatic L-amino acid decarboxylase (AADC or AAAD) (EC 4.1.1.28). Phenylethylamine undergoes *N*-methylation catalyzed by phenylethanolamine *N*-methyltransferase (PMNT) (EC 2.1.1.28) to form yet another neurotransmitter, *N*-methylphenylethylamine, in the adrenal glands (Goldstein et al., 1972) and in specific neurons in the brain (Kitahama et al., 1985). After the conversion of phenylalanine to tyrosine by AAAH, the latter is decarboxylated by AADC to form tyramine. Further reaction of tyramine catalyzed by PNMT generates N-methyltyramine; alternatively, tyramine is converted to octopamine via hydroxylation catalyzed by dopamine beta-hydroxylase (DBH)/ dopamine beta-monooxygenase (EC 1.14.17.1). If instead of decarboxylation, tyrosine is rerouted via a second AAAH reaction, the product is L-DOPA. When L-DOPA is decarboxylated by AADC, dopamine is formed. A minor pathway leads from tyramine to dopamine, with the enzyme catalyzing the hydroxylation being brain CYP2D in humans (Wang et al., 2014). Dopamine is methylated to 3-methoxytyramine by the action of catechol-*O*-methyltransferase (COMT) (EC 2.1.1.6). Dopamine is hydroxylated at the aminoethyl side chain in an *R*-specific manner by DBH to yield the major neurotransmitter norepinephrine.

After tryptophan is hydroxylated by AAAH to 5-hydroxy-tryptophan (5-HTP), following which AADC catalyzes the decarboxylation of 5-HTP into serotonin. In most animals, serotonin is found in the gastrointestinal tract (gut), blood platelets as well as in the central nervous system. It has a variety of functions in the gastrointestinal and nervous systems, a detailed description of which can be found elsewhere (Berger et al., 2009; King, 2009). Altered serotonin levels are involved in many diseases and disorders. In the liver, serotonin is oxidized by monomine oxidase to the corresponding aldehyde, which is further oxidized by aldehyde dehydrogenase to 5-hydroxyindoelacetic acid (5-HIAA), which is eliminated via urine. The amounts of serotonin and 5-HIAA are elevated in certain tumors and cancers. Serotonin is present in insect venoms, where it is the component responsible for causing pain to animals upon injection of these venoms (Chen and Larivier, 2010). Pathogenic amoebae produce serotonin, which causes diarrhea in humans (McGowan et al., 1983). Serotonin is converted by serotonin *N*-acetyl transferase to *N*-acetyl serotonin; methylation of *N*-acetyl serotonin by *S*-adenosyl methionine (SAM)-dependent hydroxyindole *O*-methyl transferase yields melatonin. Melatonin is a neuro-hormone with many functions such as antioxidant, sleep-wake regulator and immune system regulator. Tryptophan is also the source of the trace neurotransmitter tryptamine via an AADC catalyzed decarboxylation.

##### AAA decarboxylase (AADC) structure and pathology

All three decarboxylation reactions, namely, the conversion of phenylalanine into phenylethylamine, tyrosine into tyramine and tryptophan to 5-HTP have been considered to be catalyzed by the same enzyme, at least in animals. Species-specific differences between AADC produced by various organisms exist and studies in Drosophila demonstrated that different tissues may contain distinct AADC isoforms. It was shown that alternative splicing patterns from transcripts of the same gene caused the expression of tissue-specific variants (Morgan et al., 1986).

Deficiency of pyridoxine decreases AADC stability. Since, PLP is required for AADC catalysis, this is not surprising. However, the apoenzyme was found to degrade to a 20 times faster than the holoenzyme due to the involvement of a flexible loop covering the active site (Matsuda et al., 2004). The loop is fixed to the active site in the holoenzyme with a PLP Schiff base ligand interaction and stabilized; it fits into the entrance of the active site, held by hydrophobic interactions with the substrate catechol ring. The flexible loop is expected to be stabilized *in vivo* by adopting a closed structure binding the substrate aldimine, whereas the apoenzyme does not bind the substrate, leading to its preferential proteolysis (Matsuda et al., 2004). The catalytic mechanism of AADC has been postulated to involve two intermediates, a Michaelis complex followed by an external aldimine. A flexible region around the residue Arg334 is exposed before ligand binding and forms a Michaelis complex. This in turn causes a conformational change, and during the following transaldimination, a more dramatic conformational change occurs, forming an external aldimine (Ishii et al., 1998).

AADC deficiency is an inherited neuromuscular disorder in humans caused by a deficit of this enzyme. Patients show reduced catecholamine levels and elevated 3-O-methyldopa levels and have symptoms such as hypotonia, hypokinesia, and signs of autonomic dysfunction from an early age (Pons et al., 2004). 18 missense homozygous mutations have been detected by screening patients. All these mutations reduce the turnover number of the enzyme and most also alter the tertiary structure, with several experimental approaches pointing to incorrect conversion of the apoenzyme to the holoenzyme as the cause of the pathogenicity in a majority of the cases (Montioli et al., 2014).

The most striking results are observed upon mutation of the residues His70, His72, Tyr79, Phe80, Pro81, Arg447, and Arg462, which map to a key loop structure participating in the switch of the apoenzyme to the holoenzyme (Montioli et al., 2014). Mutations of Arg347 affect catalysis, while mutations of Leu38 and Ala110 cause structural/functional defects (Montioli et al., 2014).

#### The melanin pathway

Melanins are pigments that in animals are responsible for the coloration of eyes, hair, skin, fur, feathers and scales. While higher animals use melanins mainly for protection from radiation and in the immune response, insects utilize them many purposes such as hardening the cuticle, pigmentation of the exoskeleton, wound healing and innate immune responses. Melanins are derived from L-DOPA, which as mentioned before is derived from tyrosine. Tyrosinase is a rate-limiting oxidase containing copper, which catalyzes two separate steps in melanin biosynthesis, the hydroxylation of a monophenol is the first reaction, followed by conversion of the o-diphenol to the corresponding o-quinone. The o-quinone, for example dopaquinone, is further metabolized to eumelanins and pheomelanins (Solano, 2014). Dopaquinone combines with cysteine forming either 2-*S*-cysteinyl-DOPA or 5-cysteinyl-DOPA, both of which form pheomelanins via benzothiazine intermediates. In the eumelanin pathway, dopaquinone is converted to leucodopachrome, which is the parent compound for dopachrome. The next intermediate is either 5,6-dihydroxyindole (DHI) or 5,6-dihydroxyindole-2-carboxylic acid (DHICA), both derived from dopachrome. Both are converted into quinone which eventually form the eumelanins. A comprehensive review of the biosynthesis of the melanin pigments in insects and higher animals has been published elsewhere (Sugumaran and Barek, 2016).

There are multiple types of albinism caused by melanin deficiency, linked to different genes, among which type 3 oculocutaneous albinism results from a single-gene inborn metabolic defect, in this case mutations in the tyrosinase enzyme. Albinism entails a partial or complete lack of pigmentation in the skin, hair, and eyes. Albinism in humans is commonly connected with a number of vision defects and the lack of skin pigmentation may lead to heightened susceptibility to sunburn and skin cancers. Melanin granules are essential in immune cells and therefore, albinism leads to lowered immune defense (Kaplan et al., 2008). Albinism is also associated in some cases with deaf-mutism (Tietz, 1963).

##### Dopachrome converting enzymes – DCT and DCDT

All known mammalian dopachrome converting enzymes transform dopachrome into DHICA, whereas insect and invertebrate enzymes convert dopachrome into DHI. A D-dopachrome converting enzyme was first discovered in mammals (Orlow et al., 1994), which associates with tyrosinase and other enzymes in the pathway to form a melanogenic complex. It was separated from the complex, characterized (Aroca et al., 1990a,b; Pawelek, 1990) and named as dopachrome isomerase or dopachrome tautomerase (DCT) (EC 5.3.2.3). DCT is known to contain zinc at its active site (Solano et al., 1994; Furumura et al., 1998), with the metal binding site being remarkably similar to the binding site of the binuclear copper center of tyrosinase (Furumura et al., 1998). Whereas the binuclear copper is critical for oxygen activation in tyrosinase, dopachrome tautomerism does not need such chemistry. Therefore, it has been suggested that one zinc atom binds the quinonoid side of dopachrome and the other one to the imine and carboxyl groups (Palumbo et al., 1987). This geometry is considered favorable to stabilize the quinone methide intermediate and allow a rearrangement to DHICA.

The guanidium group of arginine has the ideal geometry for binding the carboxyl group of the substrate, which will stabilize the quinone methide intermediate and favor the isomerization reaction leading to DHICA production in DCT. Arg194 is modified to Gln in the *slaty* mutant and this single amino acid change is solely responsible for the drastic reduction in the enzyme activity of the enzyme (Jackson et al., 1992; Kroumpouzos et al., 1994). Therefore, Arg194 is suggested to critical to catalysis and/or substrate binding.

A dopachrome conversion factor distinct from DCT has been characterized from insects (Aso et al., 1984, 1989; Sugumaran and Semensi, 1991) and cuttlefish (Palumbo et al., 1994). Insect dopachrome conversion factor designated as dopachrome decarboxylase/tautomerase (DCDT) (EC 4.1.1.84) belongs to the yellow gene family of proteins (Drapeau, 2001). Mutation in this gene causes the color of the cuticle to be yellow to brown and hence the name (Drapeau, 2001). DCDT is linked to the innate immune response of insects (De Gregorio et al., 2001; Huang et al., 2005; Dong et al., 2006; Paskewitz and Andreev, 2008). DCDT activity is enhanced in response to microbial infections in *Drosophila melanogaster* (De Gregorio et al., 2001), while *Anopheles gambiae* infection by the malarial parasite triggers an increase in the transcripts of DCDT (Dong et al., 2006).

Unlike the DCT of mammals, DCDT exhibits promiscuous substrate specificity and attacks a number of L-dopachrome derivatives, while not converting D-dopachromes (Sugumaran and Semensi, 1991). The insect enzyme decarboxylates some dopachrome derivatives but tautomerizes others. It is unclear if DCDT contains metal cofactors, and whether it lacks the critical arginine residue found in DCT. In theory, a carboxyl residue instead of the guanidium group (from arginine) could induce the elimination of a quinone methide from the active site after dopachrome isomerization and subsequent non-enzymatic decarboxylation. If DCDT does not need metals for catalysis, then the mechanism of DCT would also have to be revised. For these reasons, solving the three-dimensional of structure of DCDT is important and would be expected to lead to major advances in understanding the melanogenic enyzmes.

#### The kynurenine pathway

**Figure 5:**
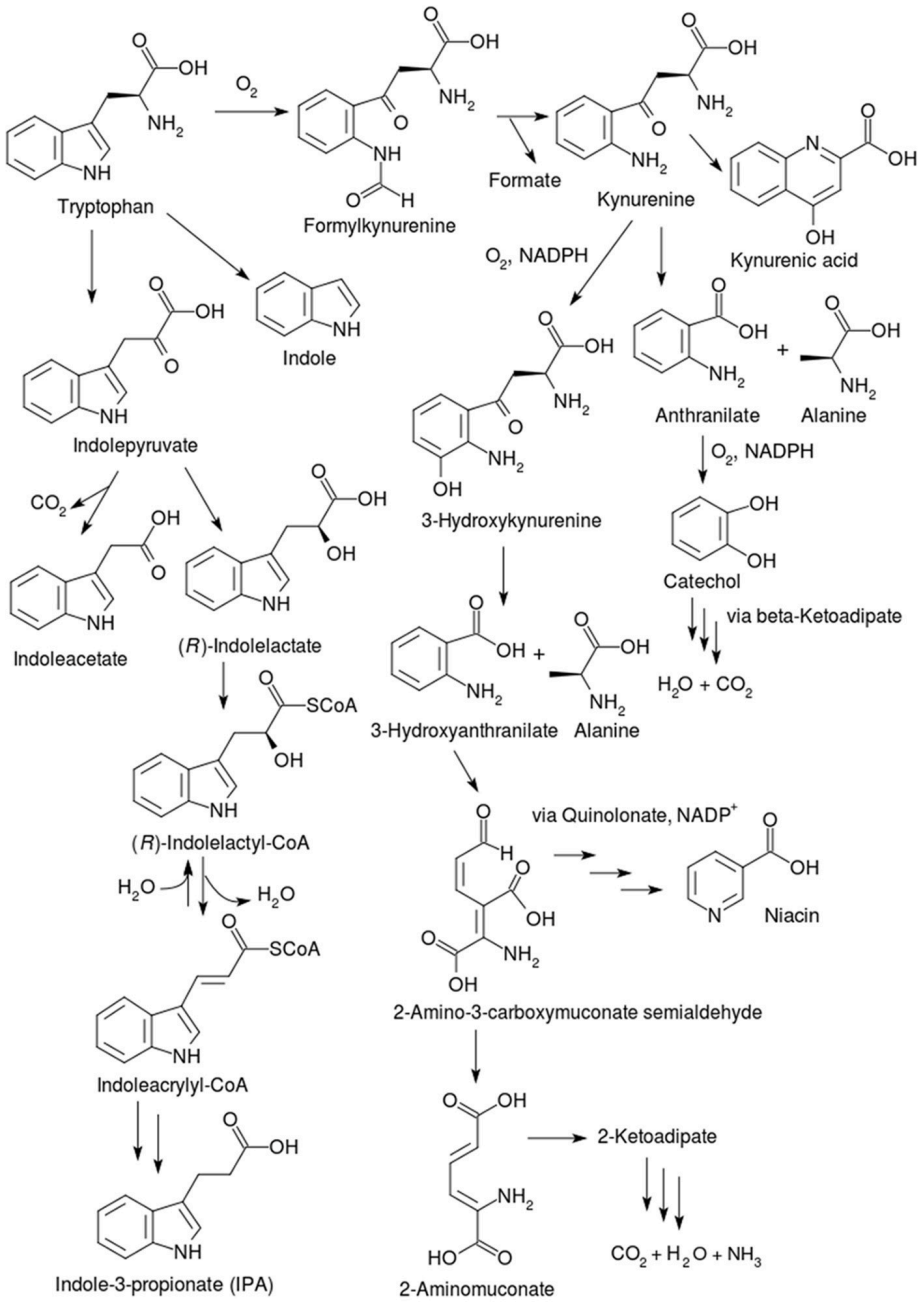
Pathways for tryptophan catabolism. The kynurenine pathways are found in different bacteria. Mammals degrade tryptophan mainly in the liver via 3-hydroxyanthranilate, while the branch proceeding via kynurenic acid is found in the brain. The other two pathways occur in gut bacteria; the generation of indole (by the action of tryptophanase) occurs in enteric bacteria, whereas indoleacetate and indolepropionate are produced via indolepyruvate in strict anaerobes, mostly *Clostridia*. Other pathways exist in *Lactobacilli* which convert tryptophan to indole-3-aldehyde (I3A). Tryptophan can also be transaminated to indolepyruvate via amino group transfer with 2-oxoglutarate or pyruvate.

This oxidative pathway for tryptophan degradation exists in both prokaryotes and eukaryotes and was first described by Kotake (1933). It is depicted on the right side of Figure 5. Over 90% of the tryptophan in the mammalian system is catabolized via this pathway, mainly in the liver, and proceeds through the intermediate 3-hydroxyanthranilate (Figure 5) and through another branch proceeding via kynurenic acid (Figure 5). Defects in this pathway in humans are implicated in serious pathological manifestations, such as Huntington's disease (Pearson and Reynolds, 1992), Alzheimer's disease (Ting et al., 2007), dementia linked to HIV (Sardar and Reynolds, 1995), and some forms of cancer (Opitz et al., 2011).

The pathway in both eukaryotes and prokaryotes starts with the oxidation of tryptophan into *N*-formylkynurenine in a heme-protein dioxygenase reaction. There are two separate heme-containing dioxygenases, typtophan-2,3-dioxygenase (TDO) (EC 1.13.11.11) and indolamine-2,3-dioxygenase (IDO) (EC 1.13.11.52), which can perform this reaction, but have low sequence homology. In mammals, the former enzyme is expressed mainly in the liver, while the IDO is found in the lungs, intestines, and brain. The next enzyme, kynurenine formamidase or arylformamidase (EC 3.5.1.9), cleaves *N*-formylkynurenine into formate and kynurenine. In eukaryotes, a mitochondrial NADPH-dependent flavoenzyme called kynurenine-3-monoxygenase (EC 1.14.13.9) hydroxylates kynurenine to 3-hydroxykynurenine. In mammals, an alternative sink for kynurenine is the formation of KA by a transamination followed by a dehydration, catalyzed by the PLP-containing kynurenine aminotransferase (KAT) (EC 2.6.1.7) in the brain. There are four different isoforms termed KAT I, II, III and IV in mammals including humans, which all have broad, but distinct substrate specificities, and have been reviewed extensively elsewhere (Han et al., 2010). KA is a non-competitive antagonist of glutamate receptors and therefore influences glutamate-mediated neurotransmission (Kessler et al., 1989). Due to this, both KA accumulation and KA deficiency have been implicated in a variety of neuropathological conditions (Schwarcz et al., 2009; Vamos et al., 2009). Apart from these, KA is an endogenous ligand for the G-protein coupled receptor GPR35 that is mainly expressed in immune cells (Wang et al., 2006) and therefore, it also participates in immune regulation. KA is also implicated in the control of cardiovascular function by affecting the appropriate areas in the medulla oblongata (Colombari et al., 2001).

Following the formation of 3-hydroxykynurenine, kynureninase or kynurenine hydrolase (EC 3.7.1.3) cleaves 3-hydroxykynurenine into 3-hydroxyanthranilate and L-alanine, in a PLP-dependent manner. Eukaryotes and many prokaryotes convert tryptophan into 3-hydroxyanthranilate by this reaction. After this stage, 3-hydroxyanthranilate is further cleaved by a non-heme iron enzyme, 3-hydroxyanthranilate 3,4-dioxygenase (EC 1.13.11.6), into 2-amino-3-carboxymuconate semialdehyde (Ichiyama et al., 1965; Zhang et al., 2005). This compound represents a branching point: it can be either catabolized via 2-aminomuconate (Martynowski et al., 2006) and 2-ketoadipate, all the way to carbon dioxide, water and ammonia, or a non-enzymatic reaction occurs, converting the 2-amino-3-carboxymuconate semialdehyde into quinolinate (Colabroy and Begley, 2005). Further reactions of quinolinate are enzyme catalyzed and lead to the formation of NAD(P)^+^ and niacin.

### AAA catabolism in microbes and implications for animal health

This section covers AAA catabolism by microflora resident in animal guts, pathogens which infect animals, as well microbes that utilize AAA for the biosynthesis of antibiotics.

#### Kynurenine pathway in microbes

While some prokaryotes share the 3-hydroxykynurenine version of the kynurenine pathway with eukaryotes including animals as discussed in the previous section, in bacteria belonging to the genus *Pseudomonas*, kynureninase preferentially acts directly on kynurenine, hydrolyzing it to anthranilate and alanine (Hayaishi and Stanier, 1952). The pseudomonads subsequently convert anthranilate to catechol by the action of an NADPH-dependent non-heme iron enzyme called anthranilate-1,2-dioxygenase (EC 1.14.12.1), thereby eliminating carbon dioxide and ammonia (Taniuchi et al., 1964). Catechol is further degraded via β-ketoadipate in several steps into water and carbon dioxide. Many bacteria and fungi employ the constitutive kynurenine pathway to furnish the essential redox cofactor NAD(P)^+^. Nevertheless, the broad distribution and inducible nature of the enzymes of this pathway among bacteria and fungi suggests roles in catabolism and secondary metabolism. Bacteria can make NAD(P)^+^ via an alternative pathway, and it has been hypothesized that the pathway from kynurenine was likely acquired by horizontal gene transfer (Lima et al., 2009). Several antibiotics can be derived from the kynurenine pathway, including sibirimycin, which arises from a modified pathway involving a methylation step (Giessen et al., 2011), and actinomycin, whose synthesis involves an additional set of some enzymes of this pathway, apart from the “normal” kynurenine pathway (Keller et al., 2010). Tryptophan dioxygenase is also implicated in the production of quinomycin antibiotics via a β-hydroxy-kynurenine intermediate (Hirose et al., 2011).

The kynurenine pathway is involved in the interplay between host and pathogens during infections. The host imposes tryptophan limitation on invading microorganisms by degrading it via the kynurenine pathway, whereas the pathogens use the pathway to synthesize compounds necessary for their growth and metabolism. Anthranilate is a key molecule which participates in many of these interactions. *P. aeruginosa* synthesizes quinolone quorum sensing molecules from anthranilate, which are important for virulence. While anthranilate can be derived from many pathways including tryptophan biosynthesis, when *P. aeruginosa* is grown in rich media, the kynurenine pathway becomes the source for anthranilate (Farrow and Pesci, 2007). Some pathogens, such as *Chlamydia psittaci*, have evolved to evade host-imposed tryptophan depletion by replacing the anthranilate synthase in the tryptophan biosynthetic operon by a kynureninase (Wood et al., 2004). Infection with *Toxoplasma gondii* has been speculated as a factor in increasing incidence of schizophrenia; in mouse models, stimulation of the kynurenine pathway was observed and the levels of several pathway metabolites including 3-hydroxykynurenine, quinolinic acid, and kynurenic acid are elevated (Notarangelo et al., 2014). During *Helicobacter pylori* infection in the human gastric mucosa, a specific up-regulation of IDO expression is observed, which regulates multiple helper T-cell lines, resulting in lowered gastric inflammation (Larussa et al., 2015), possibly directly increasing the persistence of the pathogen. The obligate intracellular pathogen, *Anaplasma phagocytophilum*, which causes one of the most common tick-borne diseases, has been shown to up-regulate the expression of a specific organic anion uptake protein and a KAT enzyme enhancing its survival in the arthropod vector (Taank et al., 2017). Recent studies have demonstrated that the pathway branching from kynurenine to kynurenic acid involving KAT is induced in bacterial meningitis and the metabolites thus produced contribute directly to the pathology of the disease (Coutinho et al., 2014).

#### AAA catabolism in gut commensals and pathogens

Aerobic, microaerophilic and strictly anaerobic microorganisms occupy the gut or gastro-intestinal (GI) tracts of animals including humans. Prominent GI tract bacteria which utilize AAA as substrates include lactobacilli, enteric bacteria and strict anaerobes (mostly Firmicutes) including the Clostridia and related genera. Microbial AAA degradation commonly involves enzymes such as aminotransferases, dehydrogenases and decarboxylases and produces products including the corresponding aromatic metabolites such as arylpyruvate, arylpropionate, aryllactate, arylacrylate and arylacetate. The levels of phenylacetate, 3-phenylpropionate and 3-phenyllactate derived from phenylalanine catabolism, and (4-hydroxyphenyl)-3-lactate from tyrosine catabolism are elevated in intestinal diseases and sepsis (Fedotcheva et al., 2008). Multiple enteric bacteria including *E. coli, Proteus vulgaris, Paracolobactrum colifome*, and *Micrococcus aerogenes* contain the enzyme tryptophanase (EC 4.1.99.1), which produces indole from tryptophan as seen in Figure 5 (Demoss and Moser, 1969). For example, Lactococci catabolize tryptophan via a tryptophan aminotransferase (EC 2.6.1.27) to IPy (Gao et al., 1997). Decarboxylases produce the corresponding primary aromatic amines from AAA (Nakazawa et al., 1977). Phenylalanine is converted into phenylpyruvate by the action of an aryllactate dehydrogenase (EC 1.1.1.110), which also accepts tryptophan as a substrate (Hummel et al., 1986). Certain unusual reactions such as the cleavage of IPy into indole and pyruvate, the conversion of tyrosine to *p*-cresol via the decarboxylation of *p*-hydroxyphenylacetate and the formation of phenol from tyrosine via the elimination of ammonia and acetate, were also detected in intestinal anaerobic bacteria (Smith and Macfarlane, 1997). The conversion of arylpyruvates into arylaldehydes is known; lactobacilli convert Ipy derived from tryptophan into indole-3-aldehyde (I3A) and phenylalanine-derived phenylpyruvate into benzaldehyde (Nierop Groot and de Bont, 1998). Perhaps the most distinctive pathway in all of AAA aerobic catabolism in the gut is found in several bacteria including *E. coli* and involves the degradation of phenylalanine via phenylacetate, through an unusual oxepin-CoA thioester synthesized by a multicomponent oxygenase, ultimately into acetyl-CoA and succinyl-CoA by means of β-oxidation (Teufel et al., 2010).

Fermentation of the AAA in the GI tract occurs in the strict anaerobes of the Firmicutes phylum. In Stickland fermentation, one AA donates electrons while another accepts them, thereby generating ATP and reducing power. Stickland electron donors include the branched chain AA, acidic AA and sulfur-containing AA as well as alanine, serine, histidine and phenylalanine; electron acceptors include glycine, proline, hydroxyproline, arginine, ornithine (derived from arginine) and tryptophan. The AAA can all be fermented as single AAs via the 2-hydroxyacid pathway, which has been studied extensively by Buckel and coworkers (Kim et al., 2004; Buckel et al., 2012). The pathway variant for AAA fermentation is termed the 3-aryllactate pathway and is discussed in detail in Radical dehydratases and the 3-aryllactate pathway. The end products of AAA reduction were known for some decades (Elsden et al., 1976). However, all the intermediates involved in both AAA oxidation and reduction in *Clostridium sporogenes* were identified much later, and the enzymes responsible for the fermentation of phenylalanine (Dickert et al., 2000, 2002), as well as of tryptophan and tyrosine (Li, 2014) were characterized in detail. Initially, the issue of whether all the AAA were catabolized via the same radical dehydratase enzymes or different ones was unresolved (Li, 2014), but later work with mutants showed that only one dehydratase was active in the degradation of all the AAA via this pathway (Dodd et al., 2017). The conversion of tryptophan to 3-indolepyruvate, oxidizing it to 3-indoleacetate, and reducing it via (*R*)-3-indolelactate and (*E*)-3-indoleacrylate to IPA, according to this pathway is depicted in Figure 5. Apart from *C. sporogenes* and *C. botulinum* (Elsden et al., 1976), recent research also uncovered other IPA producers using the same pathway in the gut, namely, *Peptostreptococcus anaerobius* CC14N and three strains of *Clostridium cadaveris* (Dodd et al., 2017).

Interest in AAA catabolism in the gut had been earlier stimulated by the fact that one of the end products of tryptophan degradation, IPA, passes through the blood-brain barrier, scavenging reactive oxygen species (ROS) in the brain by the formation of kynuric acid (Bendheim, 2002), and thereby protecting it from Alzheimer's disease (Chyan et al., 1999); it could also potentially mitigate bowel inflammation in Crohn's disease. Indeed, gut bacteria were shown to exert a large influence on the production of mammalian blood metabolites such as indoxyl sulfate and IPA; specifically IPA production was microflora-dependent and could be induced by the colonization of *C. sporogenes* (Wikoff et al., 2009). IPA is also involved in strengthening gut-barrier function by directly acting on the pregnane X receptor (PXR) (Venkatesh et al., 2014). Out of the 12 end products derived from the degradation of all the AAA via the reductive branch of this pathway, nine were detected recently in the host plasma (Dodd et al., 2017). The same authors also proposed the genetic engineering of gut bacteria involved in producing major metabolites such as IPA, as a way to influence host health.

Protozoa also occur frequently in ruminants and AAA catabolism by bacteria alone or bacteria in the presence of protozoa not only have differing utilization, but also different end products. For example, while rumen bacteria alone produced skatole, *p*-cresol and IPA as the end products, mixed bacterial-protozoan catabolism produced IAA, indolelactate and indole (Mohammed et al., 2003). Tyrosine is converted by mixed cultures of bacteria and protozoa into *p*-hydroxyphenylacetic acid and further into *p*-cresol (Mohammed et al., 2003). AAA catabolism also plays a role in bloodstream infections caused by protozoa. AAA transaminase activities were detected already in the 1950s in the pathogen which causes the leishmaniasis “kala azar”, *Leishmania donovani* (Chatterjee and Ghosh, 1957). In *Trypanosoma brucei*, the conversion of phenylalanine into phenylpyruvate and tyrosine into *p*-hydroxyphenyllactate has been reported (Stibbs and Seed, 1975a). In the same organism, tryptophan was converted into indolelactate, IAA and tryptophol (Stibbs and Seed, 1975b). The existence of AAA-related dehydrogenases, transaminases and decarboxylases was surmised from these results. Since the levels of AAA catabolites shown to originate from the pathogen were elevated in infected animals, AAA catabolism might be important for the development of sleeping sickness (Stibbs and Seed, 1975c). An AAA-dehydrogenase or L-α-hydroxy acid dehydrogenase (AHADH) has been characterized in the causative agent of Chagas disease, *T. cruzi*, whereby phenyllactate and *p*-hydroxyphenyllactate were better substrates than indolelactate (Montemartini et al., 1994).

##### Radical dehydratases and the 3-aryllactate pathway

While the oxidation of AAA catabolic pathways in aerobes and anaerobes are often similar, the reductive branches of AAA fermentations are interesting due to the unique enzymes involved. The 3-aryllactate pathway is a type of 2-hydroxyacid pathway as mentioned earlier. Phenylalanine is fermented via 3-phenyllactate, tyrosine via 4-hydroxy-3-phenyllactate and tryptophan via ILA, with all three fermentations involving catalysis by a common set of enzymes. In any 2-hydroxyacid pathway, the removal of non-acidic β-protons of 2-hydroxyacyl-CoA proceeds through a radical mechanism involving [4Fe-4S] clusters (Buckel, 2001). The first step of any AAA fermentation is the formation of the corresponding 2-ketoacid (arylpyruvate) via NAD+ and PLP-dependent transamination with 2-oxoglutarate as the amino acceptor. Then, the arylpyruvate is oxidatively decarboxylated by a pathway-specific 2-ketoacid:ferredoxin-oxidoreductase with Coenzyme A (CoA) thioesterification to the corresponding acyl-CoA (arylacetyl-CoA) which is one carbon atom shorter than the arylpyruvate. Subsequently, substrate level phosphorylation (SLP) occurs with the formation of the oxidized end product, namely the arylacetate. In the reductive branch, the 2-ketoacid is reduced by NADH-dependent *Re* (face)-stereospecific dehydrogenase (Berk et al., 1996). The resulting (*R*)-2-hydroxyacid (3-aryllactate) is thioesterified to the 2-hydroxyacyl-CoA (3-aryllactyl-CoA) by specific CoA-transferases, but the β-protons still remain unactivated (pKa ~ 40). Therefore, a special enzyme of the 2-hydroxyacyl-CoA dehydratase (2-HADH) family whose members share a common biochemical mechanism, the aryllactyl-CoA dehydratase, becomes necessary.

In every 2-HADH, a substrate radical is generated via [4Fe-4S] clusters causes Umpolung (charge reversal) at the keto group of the (*R*)-2-hydroxyacyl-CoA (Buckel and Keese, 1995). The ketyl radical generated by one-electron reduction of the substrate eliminates water via proton transfer to a conserved glutamate residue (Knauer et al., 2011). In the enoxy radical, the β-hydrogen has a pKa < 15; binding with active-site residues reduces this value further. β-Proton abstraction by the Fe-O anion from one of the [4Fe-4S] clusters generates the allylic ketyl radical, which yields the (*E*)-2-enoyl-CoA (3-arylacrylate in case of AAA degradation) and recycles an electron. Hence, the reversible α,β-*syn*-dehydration of (*R*)-2-hydroxyacyl-CoA into (*E*)-2-enoyl-CoA is a key reaction, making 2-HADH a key enzyme (Kim et al., 2004) in all variants of the 2-hydroxyacid pathway. Finally, 3-arylacrylate is reduced to the corresponding 3-arylpropionate. The reduced end product (3-arylpropionate) has the same chain length as the respective parent AAA.

**Figure 6:**
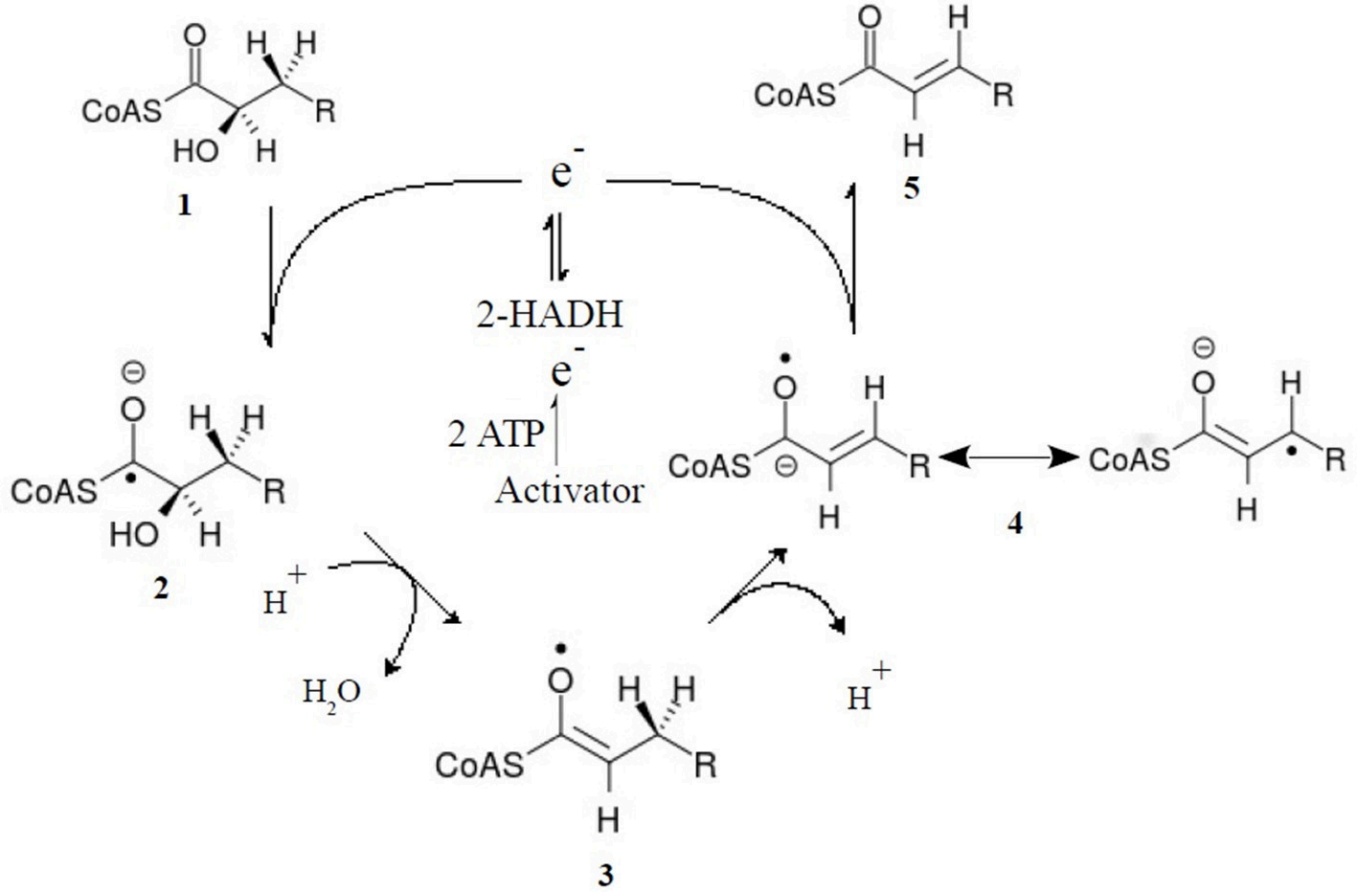
Reaction mechanism of 3-aryllactyl-CoA dehydratase. The formation of the ketyl radical anion leads to the elimination of water via proton transfer to a conserved glutamate residue and the pK_a_ of the beta-proton in the enoxy radcal is reduced from approximately 40 to about 15, a value which is further reduce by interactions with the active site residues. (1) (*R*)-2-Hydroxyacyl-CoA, (2) Ketyl radical, (3) Enoxy radical, (4) Allylic ketyl radical and (5) (*E*)-2-Enoyl-CoA.

Every 2-HADH contains two [4Fe-4S] clusters and requires reduction by a [4Fe-4S] cluster-containing ATP-ase, the activator or “archerase” (Bendrat et al., 1993). In *Clostridium sporogenes*, the aryllactyl-CoA dehydratase complex also contains the CoA-transferase (Dickert et al., 2000). One-electron reduction of a 2-HADH by its activator involves a large conformational change of the helix-[4Fe-4S] cluster-helix motif of the latter coupled to the hydrolysis of 2 ATP molecules (Knauer et al., 2011). Once the electron transfer is complete, the activator dissociates from the 2-HADH, as deduced from chelation experiments (Kim J. et al., 2008). Thus “activated,” the 2-HADH attains potentials of around −900 mV (Buckel et al., 2012) and catalyzes ~10,000 turnovers before inactivation by adventitious oxidation. Therefore, electron transfer involving [4Fe-4S] clusters as the sole cofactors in the 2-HADH-activator system facilitates radical dehydrations with minimal ATP hydrolysis. 3-Aryllactyl-CoA dehydrations are reversible, but favored in the forward direction due to the stabilization by extended conjugation afforded by the 3-arylacrylyl-CoA products (Li, 2014). The mechanism of the reaction is shown in Figure 6.

Reduced ferredoxins/flavodoxins (standard reduction potential, E0′ = −400 to −450 mV) are required (Thamer et al., 2003) to generate the reduced activators. These highly negative redox potentials could be supplied by ferredoxin- or flavodoxin-oxidoreductases which oxidize arylpyruvates coupled to the addition of CoA, yielding arylacetyl-CoA. ATP formation is coupled to the reduction of 3-arylacrylates in the 3-aryllactate pathways of *C. sporogenes* (Bader and Simon, 1983). Recent studies suggest that there is additional energy conservation in *C. sporogenes* via the translocation of Na^+^ by a *Rhodobacter* nitrogen fixation (Rnf-type) protein-complex containing the NADH:ferredoxin-oxidoreductase (Li, 2014), apart from SLP from CoA thioesters and molecular hydrogen production.

##### Type III CoA-transferase

It can be seen from the preceding section that CoA-transferases are essential for AA degradation through the 2-hydroxyacid pathways. The genes encoding the CoA-transferases are located upstream of those encoding the corresponding 2-HADH. The aryllactate CoA-transferase (FldA) from *C. sporogenes* forms a complex with the aryllactyl-CoA dehydratase (FldBC), which participates in the fermentation of AAA (Dickert et al., 2000). FldA transfers the CoA moiety from arylacrylyl-CoA to aryllactate, after which FldBC catalyzes the radical dehydration. The arylacrylyl-CoA is then converted into acrylacrylate by FldA. FldA shares 24% with CaiB from carnitine metabolism and 45% sequence identity with 2-hydroxyisocaproate CoA-transferase (HadA) involved in leucine fermentation. The three enzymes FldA, HadA and CaiB were identified as belonging to a new family of proteins called the Type III CoA-transferases (Heider, 2001). Unlike other CoA-transferases, catalysis by the Type III family entails a ternary complex mechanism without any intermediates covalently bound to the enzyme. Acid-R2 and the CoA-donor-R1 first bind non-covalently to the enzyme to form an anhydride whereby the released CoAthiolate stays at the enzyme. The attack of the CoA thiolate at the other acyl group of Acid-R2 forms the Acid-R1 and the new CoA-thioester, which are both released from the enzyme (Heider, 2001). The presence of a CoA-ligase gene in many gene clusters containing Type III transferases indicates that catalytic amounts of CoA-thioesters are required to start the reaction and prevent depletion of the CoA-thioester pool by unspecific hydrolysis.

#### AAA and the biosynthesis of antimicrobials

The three AAA, their biosynthetic precursors, as well as modified non-protein AAA are important in the synthesis of a variety of antibiotics by bacteria and fungi. Phenylalanine is incorporated into the several antibiotics, for example the bacterial cell wall biosynthesis inhibiting mureidomycins (Bugg et al., 2006) and some less common antibiotic classes such as the dithiadiketopiperazines (Brannon et al., 1971). The biosynthesis of the polyketide enterocin contains a benzoyl-CoA precursor derived from the β-oxidation of *trans*-cinnamic acid, which in turn is synthesized from phenylalanine via PAL (Piel et al., 2000; Xiang and Moore, 2002). The biosynthesis of chlorobiocin also involves 3-dimethylallyl-4-hydroxybenzoic acid, which is derived from phenylalanine by prenylation and retro-aldol condensation (Pojer et al., 2002). Tyrosine is the precursor for the biosynthesis of novobiocin, whose ring B is derived from the AAA via a coumarin intermediate (Chen and Walsh, 2001; Pacholec et al., 2005). Examples of tryptophan-derived antibiotics include actinomycin, which later became well-known as a cancer drug (Hollstein, 1974). Tryptophan-rich peptides such as indolicidin and tritrpticin, belong to a newer class of antimicrobial peptides (Chan et al., 2006), wherein this AAA has a binding preference for the interfacial regions of lipid bilayers. Combining tryptophan residues with cationic AA like arginine generates antimicrobials able to penetrate bacterial cells effectively. Recently, researchers have developed lipopeptide analogs of polymyxin B (often used in multi drug resistant cases) that incorporate tryptophan (Grau-Campistany et al., 2015).

The shikimate pathway was believed to lead to the synthesis of the intermediate amino hydroxy benzoic acid (AHBA), a compound which feeds into the biosynthesis of polyketide antibiotics of the ansamycin class, among which the anti-tubercular rifamycins are the most well-known (Sensi et al., 1959; Prelog and Oppolzer, 1973). It was later discovered that the initial compounds of the shikimate pathway, PEP and E4P are converted in a few steps into AHBA via the aminoshikimate pathway, which contains steps similar to the shikimate pathway (Ghisalba and Nüesch, 1981; Kim et al., 1996). Candicidin is an aromatic polyene macrolide antifungal molecule containing a 4-aminoacetophenone moiety (Martin and McDaniel, 1976; Martin, 1977), derived from chorismate via 4-aminobenzoic acid (PABA) by means of an aminotransferase reaction, with glutamine acting as the amino donor (Gibson et al., 1964; Huang and Gibson, 1970; Altendorf et al., 1971; Kane and O'Brien, 1975). Chorismate is converted via 4-amino-4-deoxychorismate to “p-aminophenylanine,” which then branches off into the dedicated biosynthetic pathway of the well-known antibiotic chloramphenicol (Chang et al., 2001). Chorismate is also transformed into 4-hydroxybenzoic acid in bacteria by the action of chorismate lyase which belongs to the ubiquinone biosynthetic pathway (Poon et al., 2000; Barker and Frost, 2001); 4-hydroxybenzoyl-Coenyme A has been implicated in the biosynthesis of hygromycin A (Habib et al., 2003).

Prephenate is the metabolic precursor of bacilysin produced by *Bacillus subtilis* 168, as elucidated by studies of mutants of phenylalanine and tyrosine biosynthesis (Hilton et al., 1988). Dihydrophenylalanine, a non-protein AA and antibiotic produced by *Photorhabdus luminescens*, is generated via rerouting of prephenate by the action of an unusual non-aromatizing prephenate decarboxylase, followed by a transaminase (Crawford et al., 2011). Anthranilate (formed from tryptophan degradation; Figure 5) inhibits biofilm formation by *Pseudomonas aeruginosa, Vibrio vulnificus, Bacillus subtilis, Salmonella enterica* serovar Typhimurium, and *Staphylococcus aureus*, and disrupted biofilms already formed by these bacteria via multiple mechanisms (Li et al., 2017). Therefore, anthranilate could potentially be used as a broad-spectrum biofilm inhibitor.

AAA biosynthetic precursors as well as the AAA themselves are often rerouted into the production of non-canonical AAA analogs, which form parts of antibiotic scaffolds. Obafluorin, produced by *Pseudomonas fluorescens*, is biosynthesized via the key intermediate L-aminophenylalanine (Herbert and Knaggs, 1992). Glycopeptide antibiotics such as the vancomycin and teicoplanin families, contain the non-canonical AAA analogs β-hydroxytyrosine (β-Ht), 4-hydroxyphenylglycine (Hpg) and dihydroxyphenylglycine (Dpg), all of which are capable forming rigid cross-links within the peptide. Among these, the biosynthesis of Dpg is not directly related to the shikimate pathway, but starts with the condensation of four malonyl-Coenzyme A molecules to 3,5-dihydroxyphenylacetyl-CoA (DPA-CoA) and three free coenzyme A (CoASH) (Chen et al., 2001); then, DPA-CoA is converted to 3,5-dihydroxyphenyl-glyoxylate, which is further transaminated to Dpg (Pfeifer et al., 2001; Sandercock et al., 2001).

The AAA biosynthesis intermediate prephenate is the starting point for the synthesis of Hpg, which involves the four enzymes described hence (Hubbard et al., 2000). Prephenate dehydrogenase (Pdh) converts prephenate to *p*-hydroxyphenylpyruvate, followed by 4-hydroxymandelate synthase (HmaS), which transforms *p*-hydroxyphenylpyruvate into L-*p*-hydroxymandelate and hydroxymandelate oxidase (Hmo), which oxidizes L-*p*-hydroxymandelate to *p*-hydroxylbenzoylformate. Finally, transamination of the penultimate compound by *p*-hydroxyphenylglycine transaminase (Pgat), yields Hpg. Enzymes involved in β-hydroxylation of non-ribosomal encoded amino acids were first characterized in organisms producing the antibiotics novobiocin and nikkomycin (Chen and Walsh, 2001; Chen et al., 2002). Vancomycin biosynthesis involves similar enzymes. Tyrosine is first activated to a thiol ester and attached to one of the modular thioesterease enzyme domains for antibiotic synthesis. The thiol ester is oxidized by an oxygenase which adds a β-hydroxyl-group while the substrate is still attached to the thioesterase domain, and finally, Bht is released from the module (Donadio et al., 2005). Chloro-β-hydroxytyrosine is also found in some glycopeptide antibiotics, but the chlorination time point was demonstrated to be later than the stage of free Bht synthesis (Puk et al., 2004) and is hypothesized to be during the stage of heptapeptide synthesis.

## Conclusions

The metabolism of the AAA pathways offer a rich source for the understanding of many aspects of plant, animal and microbial metabolism. Research in this area affords the opportunity to further investigate the involvement of the AAA and their metabolite derivatives in a variety of functions and roles related to the health of plants and animals. There are many aspects pertaining to the regulation, role, and function of enzymes involved in the anabolism or catabolism of compounds derived from the AAA. In addition, there are many structural aspects of key enzymes involved in these pathways that have yet to be elucidated.

## Author contributions

All authors listed have made a substantial, direct and intellectual contribution to the work, and approved it for publication.

### Conflict of interest statement

The authors declare that the research was conducted in the absence of any commercial or financial relationships that could be construed as a potential conflict of interest.

## Footnotes

^1^Database of essential genes: http://www.essentialgene.org/

^2^PAH Mutation Analysis Consortium database: http://www.mcgill.ca/pahdb
